# Leveraging autocatalytic reactions for chemical domain image classification†

**DOI:** 10.1039/d0sc05860b

**Published:** 2021-03-03

**Authors:** Christopher E. Arcadia, Amanda Dombroski, Kady Oakley, Shui Ling Chen, Hokchhay Tann, Christopher Rose, Eunsuk Kim, Sherief Reda, Brenda M. Rubenstein, Jacob K. Rosenstein

**Affiliations:** School of Engineering, Brown University Providence RI USA jacob_rosenstein@brown.edu; Department of Chemistry, Brown University Providence RI USA

## Abstract

Autocatalysis is fundamental to many biological processes, and kinetic models of autocatalytic reactions have mathematical forms similar to activation functions used in artificial neural networks. Inspired by these similarities, we use an autocatalytic reaction, the copper-catalyzed azide–alkyne cycloaddition, to perform digital image recognition tasks. Images are encoded in the concentration of a catalyst across an array of liquid samples, and the classification is performed with a sequence of automated fluid transfers. The outputs of the operations are monitored using UV-vis spectroscopy. The growing interest in molecular information storage suggests that methods for computing in chemistry will become increasingly important for querying and manipulating molecular memory.

## Abstract 2

Kinetic models of autocatalytic reactions have mathematical forms similar to activation functions used in artificial neural networks. Inspired by these similarities, we use a copper-catalyzed reaction to perform digital image recognition tasks.

## Graphical abstract

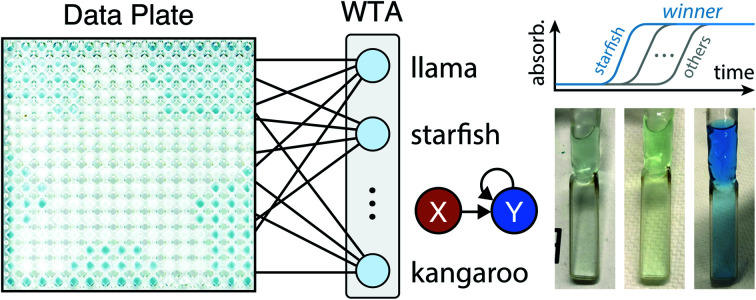

## Introduction

An autocatalytic reaction is one which is catalyzed by its own products. Such reactions can exhibit interesting behaviors such as self-sustaining growth and oscillation, and play important roles in living systems.^1^ Autocatalysis occurs in elements of cellular metabolism including glycolysis,^2^ mitosis,^3^ apoptosis,^4^ and DNA replication.^5^ Some have even posited that the origin of life may have had connections to the emergence of autocatalytic networks.^6,7^

The dynamics of autocatalytic reactions share some features with modern machine learning algorithms, in which cascades of nonlinear operators are used to efficiently realize functions of arbitrary complexity.^8^ In theory, a network of autocatalytic reactions can be made analogous to an artificial neural network.^9^ Moreover, autocatalytic reactions have the beneficial property that their inputs and outputs can be represented by the same chemical species, potentially offering experimental scalability for deep feedforward networks.

The idea of chemical computing has a long history, inspired in part by the power, complexity, and energy efficiency of living systems.^10,11^ Recent advances in molecular information storage^12–17^ have brought these unconventional systems closer to reality and have renewed interest in chemical computing. Much of the research on molecular computing has focused on *in vitro* gene expression circuits^18,19^ and DNA strand–displacement reactions.^20–22^ While genomic networks have important experimental advantages, such as well-established catalytic enzymes, they represent a narrow slice of chemical space, and hybridization-based computation often suffers from slow reaction rates and temperature sensitivity. Outside of DNA, chemical computation has been demonstrated using oscillating reactions,^23,24^ metabolites,^25^ and phenols,^26^ and has been the subject of many theoretical studies. *In silico* chemical reaction networks^27^ have been designed to not only implement feedforward neural networks,^28,29^ but to both train and execute learned functions in simple perceptrons.^30,31^

We previously demonstrated a chemical perceptron which performs parallel computations on several datasets encoded in the co-existing concentrations of different chemical species.^26^ Using this system, we classified several handwritten digits from the MNIST database.^32^ However, this classifier was based on volumetric transfers of unreactive species, which amount to linear operations in the chemical domain. As a result, the final threshold operation had to be performed *in silico*.

Here, we combine automated fluid handling with an autocatalytic reaction to realize nonlinear operations *in chemico*. We encode digital images into catalyst concentrations, apply linear multiply-accumulate operations using volumetric liquid transfers, and perform winner-take-all (WTA) image classification with autocatalytic reactions. These demonstrations are a promising step in the nascent development of synthetic chemical computing systems.

## Results and discussion

### Kinetics of autocatalysis

**Fig. 1:**
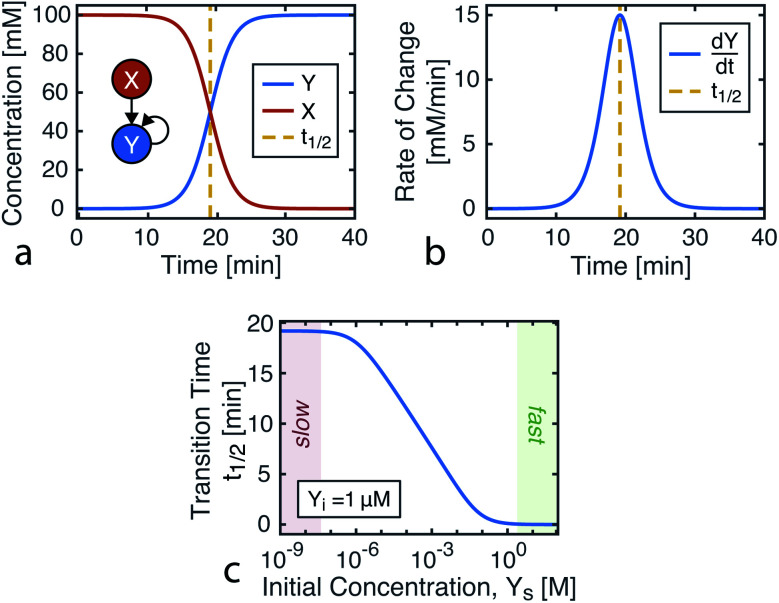
Kinetics of autocatalysis. (a) Reagent and autocatalytic product evolution over time for *X*_o_ = 100 mM, *Y*_o_ = 1 μM, and *k* = 0.1 (eqn (4)). (b) Rate of product concentration change over time for the reaction simulated in a, showing the accelerated production typical of an autocatalytic process. (c) The time to transition as the input catalyst concentration (*Y*_o_) is varied about a constant: *Y*_o_ = *Y*_i_ + *Y*_s_ with *Y*_i_ = 1 μM, *X*_o_ = 100 mM, and *k* = 0.1.

A reaction in which one of the products speeds up further product formation is called autocatalytic. Consider the simplest autocatalytic reaction, which is given by:^33^1
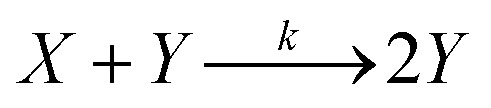
and has the following first order rate law:2
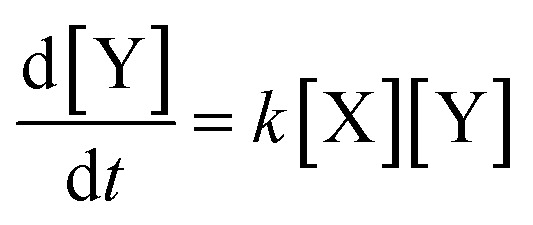
Since mass must be conserved, and the volume of dilute reaction solutions is approximately constant, the sum of the initial concentrations (*X*_o_ and *Y*_o_) must equal the sum of the concentrations at any time: *X*_o_ + *Y*_o_ = [X] + [Y]. Applying this conservation law reduces the differential equation to a single variable:3
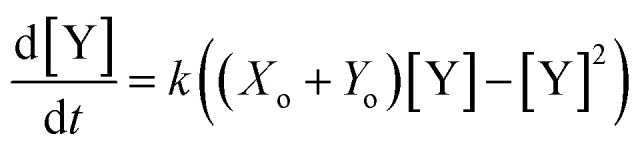
which can be solved *via* integration to obtain an expression for the product evolution as a function of time:4
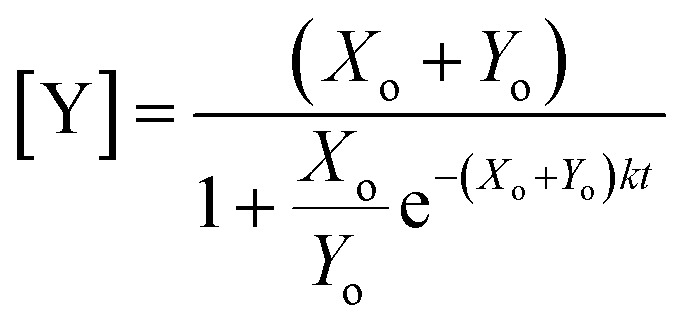
Plotting eqn (4), we can see that the catalytic product evolution follows a sigmoidal trajectory (Fig. 1). Initially, there is a slow accumulation of the catalytic species *Y*. When enough catalyst has formed, product formation accelerates until the limiting reagent (*X*) is consumed, and the output concentration settles to a constant final value.

### Relationship to artificial neural networks

**Fig. 2:**
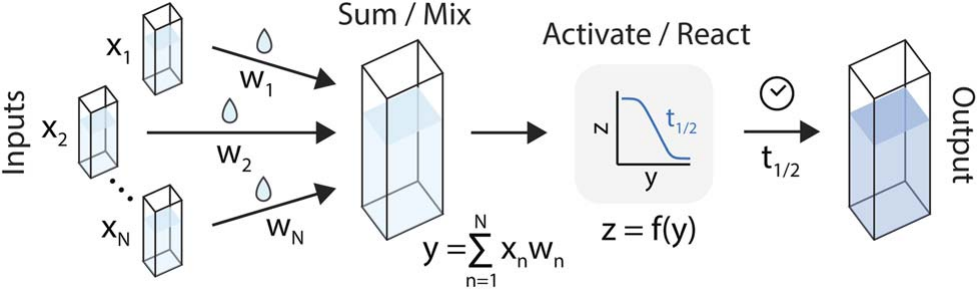
An artificial neuron implemented in the chemical domain through the programmable mixing and reacting of compounds from an autocatalytic process.

An artificial neuron is a basic learning unit, inspired by biological neurons, which multiplies its inputs by a set of weights and transforms their sum through a nonlinear operator (the ‘activation function’).^34^ Interconnected sets of artificial neurons can perform classification tasks, among many other applications. In theory, the nonlinear kinetics of autocatalytic reactions could suggest chemical equivalents to electronic artificial neurons (Fig. 2).

For instance, the product evolution curve from eqn (4) is analogous to the popular logistic activation function.^35^ However, controlling this type of reaction through timing would be experimentally challenging. Previous theoretical work^9^ instead added a feedback path with a reverse reaction (*Y* back to *X*) with different kinetics. In this arrangement, the final product concentration ([Y]_*t*→∞_) was either a constant or zero, depending on whether the forward or reverse reactions were dominant. This network effectively produced a rectifying activation function.^36^ While simulations show this design could be robust against large concentration variations, it would be quite challenging to implement since it requires many complementary autocatalytic reactions with programmable reaction rates and limited cross-reactivity.

Taking into account experimental constraints, here we structure chemical computations around the time it takes for the product to evolve, using a single autocatalytic reaction as a nonlinear programmable time delay generator. In this model, the initial conditions are the input variable and the time to transition is the output (Fig. 1c). By using one reaction under varying initial conditions, data from a simple dilution ladder can provide sufficient calibration to design a computational network.

### Time to transition

We define the time to transition, *t*_1/2_, as the time at which the product concentration, [Y], is halfway between its initial and final values:5
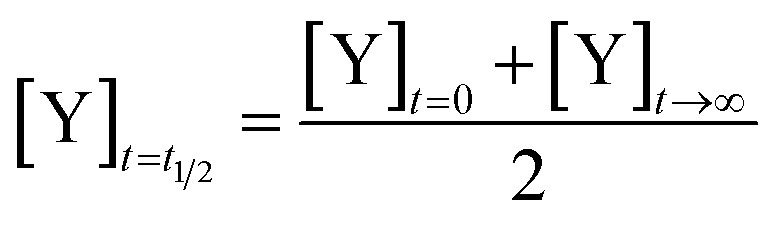
For the reaction described by eqn (4), the time to transition is given by:6
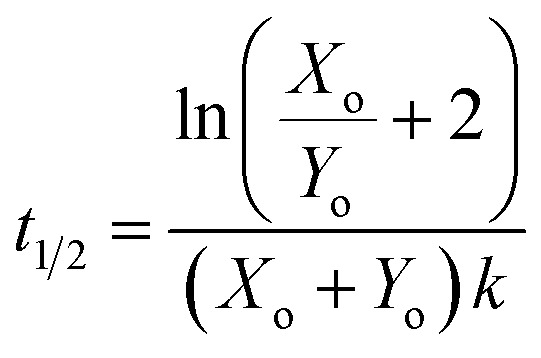
Fig. 1c shows how this transition time varies with initial catalyst concentration. The transition times are bounded by two extremes: the slow regime (left) where the amount of added catalyst is too little to speed up the reaction and the fast regime (right) where the catalyst accumulation is no longer the limiting step of the reaction.

For this reaction, the rate of increase in catalyst (d[Y]/d*t*) is greatest at *t*_p_ = ln(*X*_o_/*Y*_o_)/((*X*_o_ + *Y*_o_)*k*) which, assuming the initial concentration ratio is large (*X*_o_/*Y*_o_ ≫ 2) occurs roughly at the time to transition (*t*_p_ ≈ *t*_1/2_). Using either of these time points as the output parameter, instead of the final concentration ([Y]_*t*→∞_), makes for more consistent computations since the final concentration tends to be more variable than the timing of catalysis (see Fig. 8c).

### Copper-catalyzed Azide–Alkyne cycloaddition

**Fig. 3:**
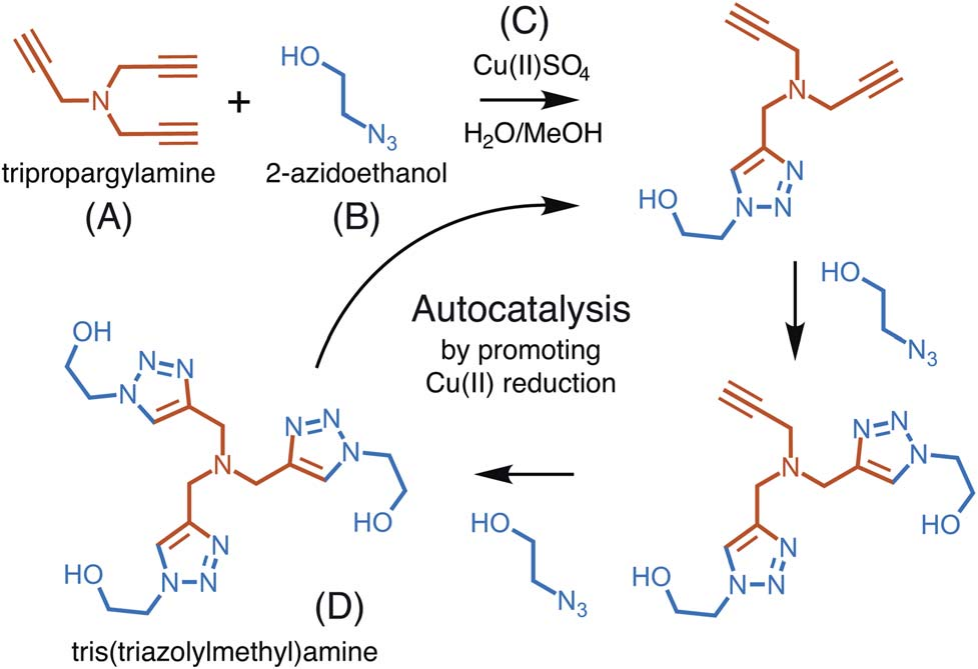
An overview of the copper (C) catalyzed azide–alkyne cycloaddition reaction, showing the buildup of triazole branches on the amine backbone of (A) after each azide (B) incorporation. The three-branched product (D) catalyzes its own generation by promoting the reduction of Cu(ii).

The copper-catalyzed reaction of an azide and an alkyne to form a 5-membered ring containing heteroatoms, known as a triazole, is one of the most well studied click reactions.^37^ These reactions have fast kinetics, are irreversible, use readily available starting material, occur under mild conditions, are high yield, and do not require purification.^38^ One such copper-catalyzed azide–alkyne cycloaddition (CuAAC) reaction was recently shown to exhibit particularly strong, autocatalytic rate enhancement.^39^ Carried out in a water–methanol solution containing a dissolved copper(ii) salt, the reaction occurs through 1,3-cycloaddition and uses one equivalent of an alkyne, tripropargylamine, and three equivalents of an azide, 2-azidoethanol, to form a final product, tris(triazolylmethyl)amine, composed of three triazoles (Fig. 3).

Rate enhancement was originally thought to be due to the formation of an intermediate which promotes the reduction of copper(ii) to copper(i), a common catalyst.^40^ However, it has now been shown that the final product in complex with copper(i) is a more reactive catalyst for cycloaddition than copper(i) alone.^41^ The formation of tris(triazolylmethyl)amine increases copper(i) production and activity, thereby increasing its own formation and resulting in autocatalysis.

### Monitoring reaction progress

**Fig. 4:**
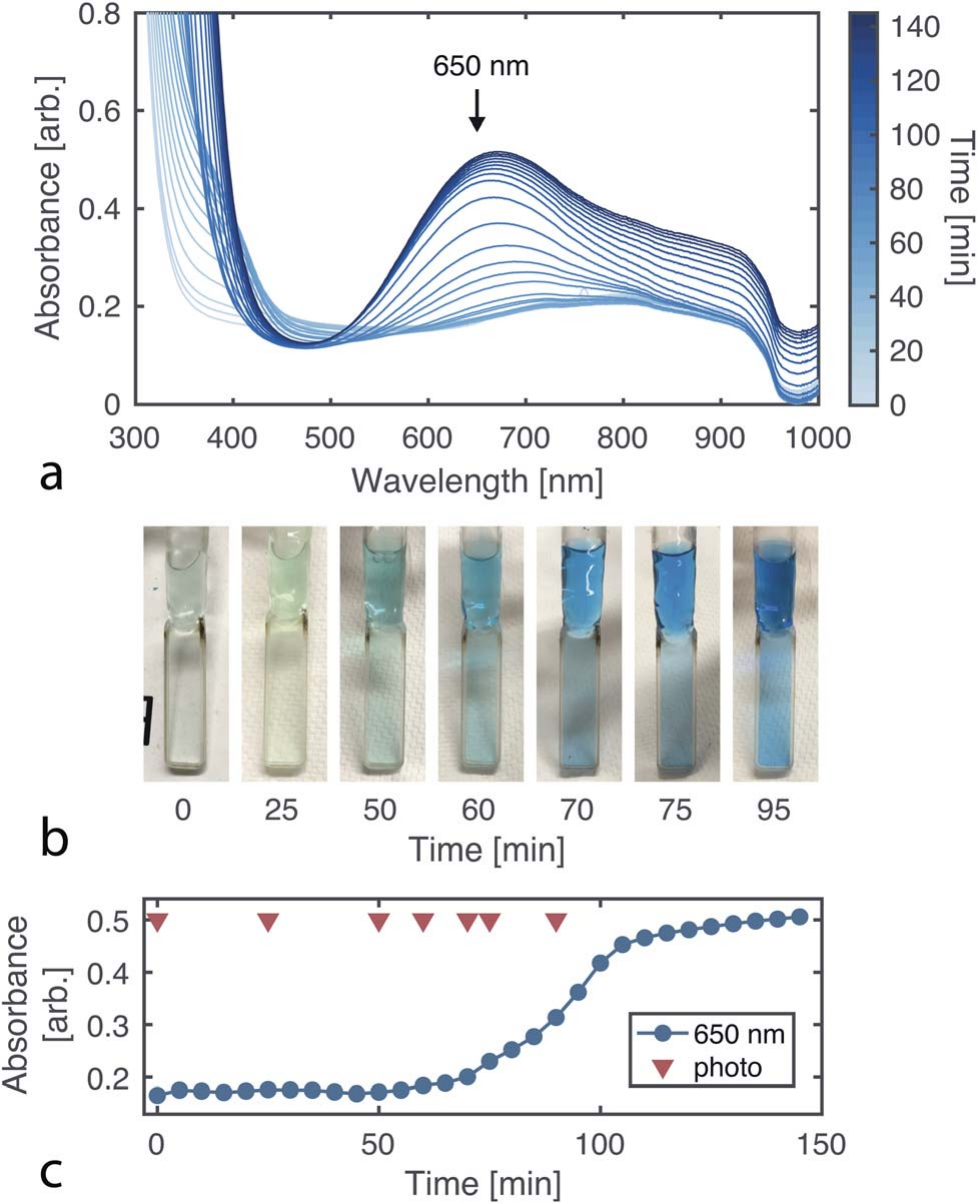
Monitoring reaction progress *via* UV-vis absorbance. (a) Absorbance spectra over the course of the CuAAC reaction (sampled every 5 min for 145 min). (b) Images of the reaction solution over time, showing the color transition as product is formed. (c) Absorbance time series for 650 nm light. The product is known to absorb at this wavelength. The reaction was run with 102 mM tripropargylamine, 290 mM 2-azidoethanol, and 40 mM CuSO_4_ in 94% deionized water and 6% methanol. The UV-vis measurements were taken with the Varian Cary 50 Spectrophotometer using a Schlenk quartz cell with a pathlength of 2 mm.

Since the CuAAC reaction involves multiple copper–ligand complexes which absorb visible light, we can quantitatively monitor reaction progress using UV-vis spectroscopy. Fig. 4a plots the reaction progression, with the broad absorption at 650 nm corresponding to the copper(ii) complexes of triazolylmethylamine.^39^

To initially validate the reaction, we carried out CuAAC reactions in cuvettes, allowing 290 mM 2-azidoethanol, 102 mM tripropargylamine, and 40 mM copper(ii) sulfate to react while product formation was monitored using a UV-vis spectrophotometer (Varian Cary 50). The initial solution is transparent and colorless while the final solution containing the tris(triazolylmethyl)amine product has a blue tint (Fig. 4b).

For subsequent high-throughput experiments, we adapted the reaction to 384-well plates, using a UV-vis microplate reader (BioTek Synergy HTX) to track product formation.

### Reaction parametrization

The CuAAC reaction time can be programmed by seeding the reaction with a small amount of tris(triazoloylmethyl)amine. The time to half completion (*t*_1/2_) is a function of the initial reagent concentrations ([A]_*t*=0_, [B]_*t*=0_, [C]_*t*=0_) and seed catalyst concentration ([D]_*t*=0_). By holding the starting reagent concentrations constant ([A]_*t*=0_ = 320.6 mM, [B]_*t*=0_ = 908.8 mM, and [C]_*t*=0_ = 126.5 mM), the catalyst concentration is made the only free variable.

For the purposes of capturing the completion rate dependence on catalyst concentration, rather than developing a new system of differential equations to specifically model the CuAAC reaction, we can use eqn (6) as an intuitive template. By setting *X*_o_ = α and *Y*_o_ = *β* + [D]_*t*=0_, we arrive at a parametric equation for the time to transition when only the catalyst concentration is varying:7
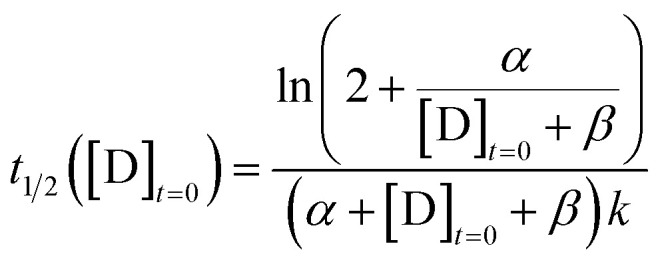


To obtain the catalyst ([D]_*t*=0_) for seeding the reaction, we pre-react a concentrated mixture ([A]_*t*=0_ = 1.308 M, [B]_*t*=0_ = 3.708 M, and [C]_*t*=0_ = 0.516 M) of reagents for 48 hours. For simplicity, we assume the reaction runs to completion, yielding a product concentration of 1.236 M, which is one third the concentration of the limiting reagent, the azide.

### Transition time calibration

**Fig. 5:**
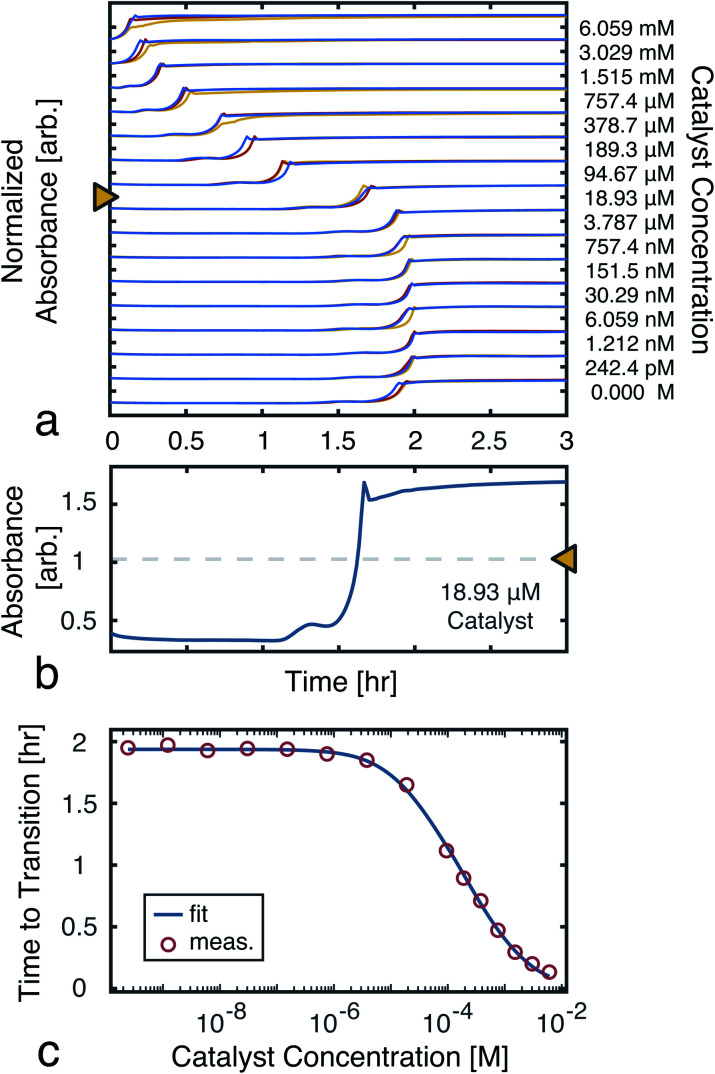
Time to transition calibration. (a) Absorbance (650 nm) traces used to monitor reaction progress as seed catalyst concentration was varied. The reactions were performed in triplicate to ensure the results were consistent. (b) A single trace from a ([D]_*t*=0_ = 18.93 μM), showing the mid-point line used to find the transition time. (c) Measured time to transitions for each tested catalyst concentration (averaged over the triplicates). A fit to eqn (7) is also shown. The model parameters were found, using nonlinear least-squares, to be: *α* = 2.81 mM, *β* = 14.2 μM, and *k* = 970 M^−1^ h^−1^.

To model the constrained CuAAC, we performed a series of reactions with varying seed catalyst concentrations. Starting with concentrated pre-reacted solution (containing about 1.2 M of catalyst), we performed serial dilutions in 94% water and 6% methanol. Samples of 1 μL from each diluted catalyst solution were then transferred to a 384-well plate. In each well, 50 μL of starting reagent solution was added to initiate the reaction. Once the transfers were completed, the plate was placed in a UV-vis plate reader to obtain the absorbance traces shown in Fig. 5a.

From the UV-vis traces, we extracted the time to transitions as the time points at which product absorbances were halfway between their initial and final values (Fig. 5b). Based on these curves, we fit a model of the transition time, which was in turn used for simulations and experiment planning (Fig. 5c).

### Winner-take-all network

**Fig. 6:**
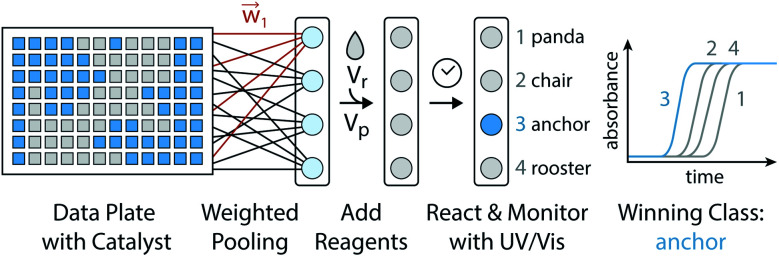
Experimental setup for evaluating a chemical WTA network. A binary image data plate is made using seed catalyst. The wells of this plate are volumetrically weighted and summed together into pools associated with each considered class (*e.g.* panda, chair, anchor, or rooster). Reagents for the CuAAC reaction are added to these pools, which are placed on a UV-vis plate reader to monitor the progression of the reactions. The class whose reaction reaches half completion first is declared the winner and assigned to the input data.

One algorithm suitable for reaction-based time delays is a winner-take-all (WTA) neural network.^42^ Such a network can be thought of as a race between potential classes, where the first class to reach a target state is deemed the winner. A diagram of a representative WTA network is shown in Fig. 8b, where each of the pooled outputs, *y*_*k*_, are associated with a different class. A comparison between the pools is used to determine the class of the input data (*x⃑*). Despite their relative simplicity, these networks can be designed to efficiently approximate any continuous function.^43^ Here, we set out to implement a chemical WTA network for image classification. An overview of the proposed computing framework is shown in Fig. 6.

### Encoding data in catalyst concentration

Digital images are represented chemically by the initial concentration of catalyst. Each pixel in a binary input image (*x⃑*) is assigned a position on a well plate, and an initial volume of solvent (*V*_s_) is added to each of these wells. Then, for each white pixel (*x*_*n*_ = 1), a small volume of pre-reacted solution (*V*_d_), with a catalyst concentration *D*_o_, is added to its well. No catalyst is added to wells corresponding to black pixels (*x*_*n*_ = 0). Thus the final catalyst concentration in data well *n* will be:8
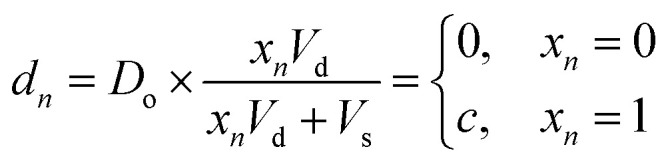
where *c* = *D*_o_ × *V*_d_/(*V*_d_ + *V*_s_) is the nonzero concentration of catalyst associated with white pixels. While more bits could be represented per well by allowing intermediate concentration levels (*e.g. c*/2, *c*/4, *c*/8), for these demonstrations we elected to use a dataset of binary images.

### In-solution multiply and accumulate

The network inputs are mapped to class-specific pools through volumetric multiply-accumulate (MAC) operations.^26^ A small volume, *v*_*kn*_, is sampled from each of the *n* data wells and transferred to pool *k*. The amount taken from each well is set as *v*_*kn*_ = *w*_*kn*_ × *V*_u_, where *V*_u_ is the maximum volume allowed to be transferred from a well and *w*_*kn*_ ∈ [0, 1] is a tuning factor. By scaling the transfer volumes with weights trained on class *k* (*w⃑*_k_), the summed output pool (*y*_*k*_) can be made to represent a single MAC operation on the catalyst-encoded input data (*d⃑*). The resulting catalyst concentration in class *k*'s pool is given by:9
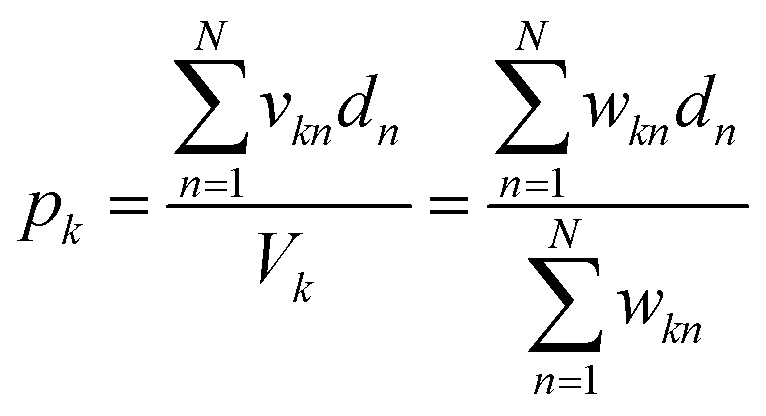
where the final volume in the pool is given by: 
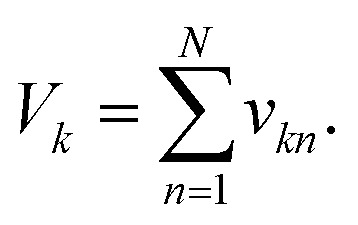
 This operation is repeated for each class, producing *K* output pools. At least *V*_s_/*V*_u_ such pools could be made from a single data plate.

### Autocatalytic activation and classification

Each of the MAC pools are composed solely of solvent and diluted catalyst, and as such only represent a linear combination of the inputs. To obtain the nonlinear response desired for classification, the autocatalytic reaction has to be initiated. To do so, a small volume, *V*_p_, is transferred from each of the pools wells to their corresponding reaction wells, which were prefilled with *V*_r_ of starting reagent solution. At first, the seed catalyst concentration in reaction well k will be given by:10
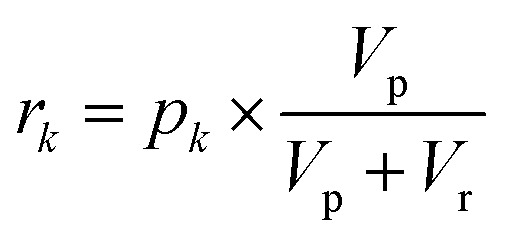
but the catalyst concentration will increase as the reaction proceeds. If we use the same conditions as the calibration experiment (Fig. 5), namely the same reagent concentrations and a volume ratio *V*_p_ : *V*_r_ roughly equal to 1 : 50, then the time at which the catalyst concentration in well *k* reaches half its steady state value will be:

11 *τ*_*k*_ = *t*_1/2_(*r*_*k*_)

where *t*_1/2_ is the time to transition model from eqn (7) and Fig. 5c. If the pooling weights are tuned such that the first reaction well to run to half completion represents the class most similar to the input data (see Methods), then we can assign class *i* to the input when:

12 *τ*_*i*_ < *τ*_*j*_, ∀*i* ≠ *j*

where the transition times can be found by monitoring the reaction wells with a UV-vis plate reader.

### Experimental demonstration

Using the proposed approach, we built an autocatalytic WTA network for classifying binary images from the CalTech 101 16 × 16 Silhouettes dataset^44^ (Fig. S1 and S2†). The network was specifically designed to identify five (*K* = 5) of the more recognizable image classes: ‘starfish’, ‘kangaroo’, ‘llama’, ‘dragonfly’, and ‘ibis’ (68–86 images per class, shown in Fig. S3–S7†).

**Fig. 7:**
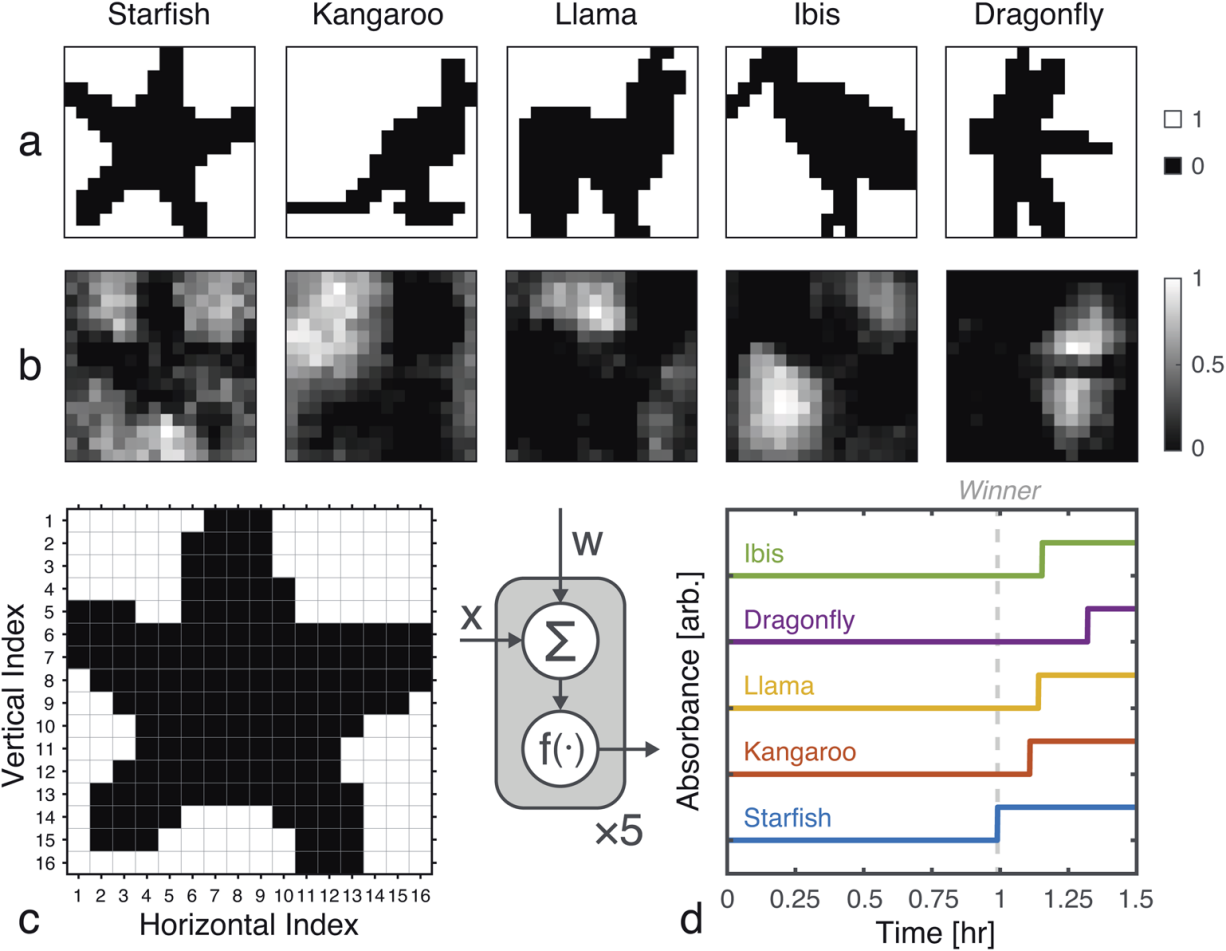
Network training and *in silico* simulation. (a) Example images from each of the considered classes. (b) Trained weights for each class. (c) The input, one of the starfish images from the test set, prior to being reshaped as a feature vector. (d) Simulation results obtained using the weights shown in (b), the image from (c), and the time to transition model from Fig. 5c.

Network weights were iteratively trained over 700 epochs, using a 70–30% train-test split (Fig. S8†). The training algorithm is described in the Methods, and the resulting weights are shown graphically in Fig. 7, alongside an example image from each class. Using these weights and the time to transition model from Fig. 5, we simulated the outputs of the network for one input image (Fig. 7c and d). Extending these simulations to the full train and test sets (398 images in total), the network was found to have a classification accuracy of 81.16% (Fig. S9†).

**Fig. 8:**
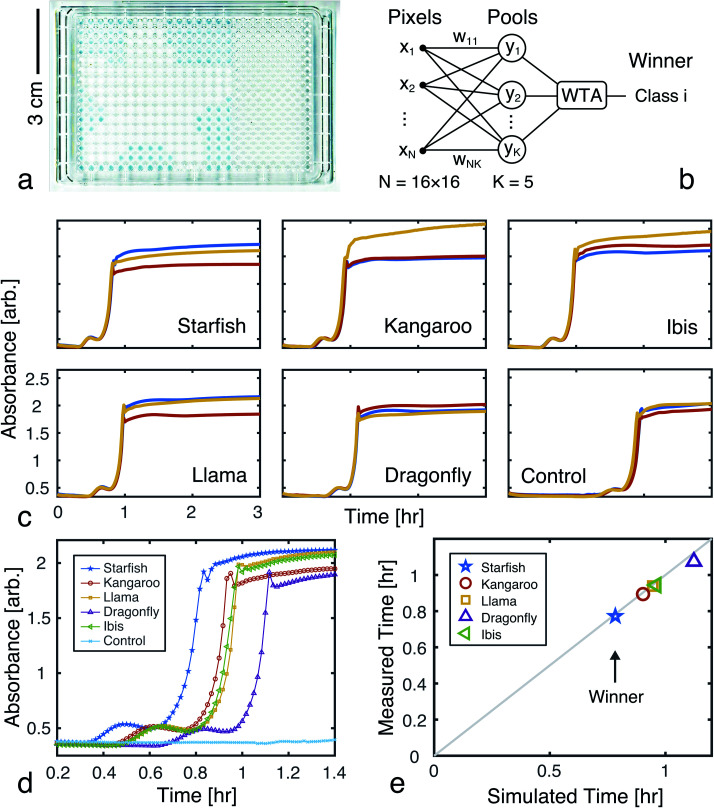
Experimental demonstration of chemical image classification. (a) Image of the data plate, containing a binary image of a starfish represented in the presence (blue, 200 nL added) or absence (transparent, 0 nL added) of pre-reacted catalyst (nominally 618 mM) in solvent filled wells (9.5 μL of 94% deionized water and 6% methanol). High pixels (*x*_*n*_ = 1) contain a presumed catalyst concentration of 12.742 mM, while low pixels (*x*_*n*_ = 0) have 0 mM of catalyst. (b) Diagram of the winner-take-all classification network implemented in liquid-phase autocatalytic chemistry. (c) Pooled well absorbance traces (each repeated in triplicate) for the 5-class WTA network. (d) Overlay of the absorbance traces for each class' first pool, showing the winner to correctly be the “Starfish.” (e) The mean measured and simulated times to transition for each of the pooled wells. Simulated data was offset by −12.162 minutes, which was the amount of time it took between introducing the reagent solution and the start of plate monitoring.

A 256-pixel binary image of a starfish, shown in Fig. 7c, was written to a 384-well plate using catalyst presence/absence encoding. The data plate preparation began by first dispensing *V*_s_ = 9.5 μL of solvent (6% methanol in water) to all wells. Wells corresponding to white pixels (*x*_*n*_ = 1) received an additional *V*_d_ = 200 nL of 2×-diluted pre-reacted solution, nominally containing *D*_o_ = 618 mM of catalyst. Wells corresponding to black pixels (*x*_*n*_ = 0) had no catalyst added. An image of the resulting data plate is shown in Fig. 8a, where a faint blue color can be seen in the wells that contain catalyst (*c* = 12.742 mM).

The weights for each considered class were applied to the starfish image, resulting in 5 separate pools. A small volume (*V*_p_ = 1 μL) from each output pool was transferred, in triplicate, to wells on a new 384-well plate. To begin the CuAAC reactions, *V*_r_ = 50 μL of starting reagent solution was added to each well. The plate was promptly loaded into a UV-vis reader to track the progression of the reactions.

Absorbance measurements were taken every 60 seconds over the course of 8 hours. The measured signals are shown in Fig. 8c and compared in Fig. 8d. Consistent with the simulation, the ‘starfish’ pool was the first reaction to complete. The runner up, kangaroo, was 6.5 minutes behind. On average, measured transition times deviated from simulations by 2% (Fig. 8e).

### Perspectives for chemical computing

DNA has often been the chemistry of choice for many developments in chemical computing,^10,21,22,45–50^ and it will continue to provide a powerful foundation for molecular-scale computation. DNA reaction networks benefit from the sequence-specificity of hybridization reactions and the availability of numerous catalytic enzymes. Strand displacement reactions, for instance, have been used to perform winner-take-all classification of 10 × 10 pixel binary images.^22^ However, because the operations rely on specific DNA sequences, a relatively large number of reagents must be designed and synthesized *a priori*. In contrast, the CuAAC reaction requires only three inputs, allowing for rapid re-configuration. Furthermore, DNA is often constrained to biologically relevant conditions (*e.g.* pH, temperature, salinity). Opportunities to utilize a broader range of catalytic reactions, which use fewer reagents or operate outside of physiological conditions, may open up new avenues for chemical computing.

Autocatalytic processes, with their nonlinear response and input–output self-similarity, represent an attractive substrate for chemical based computing. In this work, we have shown, with a simple single-layer WTA network, how the kinetics of an autocatalytic reaction could be exploited for *in chemico* image classification. In the future, autocatalytic computation could be extended to more complicated architectures, such as a multi-layered WTA network that uses multiple rounds of volumetric pooling. Because the CuAAC reaction progress can be monitored through visible color changes there is no need for additional reporter molecules. Additionally, since the CuAAC reaction involves a reactive copper species (Cu(i)), it could be coupled to other reactions which would influence triazole production, potentially allowing for more complex and dynamic operations.

## Conclusions

In summary, we designed and implemented a simple neural network using an array of autocatalytic reactions. This research extends previous chemical computing efforts which utilized non-reactive mixtures and were limited to linear mathematical operations.^26^ Here, we adapted the autocatalytic CuAAC reaction to serve as the nonlinear activation function needed for WTA classification. This work represents unique experimental progress towards a fully liquid-phase chemical classifier, in a non-biological chemistry. We anticipate that autocatalytic reaction networks will play a critical role in the future development of advanced chemical-domain computing systems.

## Methods

### Materials and reagents

All solutions were prepared using in-house deionized water (Millipore Milli-Q), having a resistivity of 18.2 MΩ cm at 25 °C, and HPLC-grade methanol (>99%, Fisher Scientific, Waltham, MA). The CuAAC reaction was done, in a solvent comprised of 6% methanol and 94% water, using 2-azidoethanol (98%, Santa Cruz Biotechnology Inc, Dallas, TX) as the azide, tripropargylamine (98%, Sigma Aldrich, Natick, MA) as the alkyne, and copper(ii) sulfate (>99%, Sigma Aldrich, Natick, MA) as the source of copper ions. The reactions were conducted at room temperature, in sealed well plates and cuvettes to prevent evaporation. Low dead volume 384-well microplates made of cyclic olefin copolymer (LP-0200, Labcyte Echo) and 384-well microplates made of polypropylene were used for high throughput experiments (PP-0200, Labcyte Echo).

### Instrumentation and analysis

An Echo 550 (Labcyte) acoustic fluid handler was used to perform the volume transfers for the high throughput experiments. Custom Python scripts were used to generate fluid handling instructions. Individual UV-vis measurements were taken with the Cary 50 (Varian), while the Synergy HTX (BioTek) platereader was used for arrayed measurements. Custom MATLAB scripts were used for network design, data analysis, and visualization.

### Network training

The objective of training is to produce a matrix of weights which maximize the accuracy of the WTA network. In order to correctly identify an image, the pool for its associated class should transition before that of any other class. Because volumetric multiply-accumulate operations correspond to positive weights^26^ and since there is an imposed upper limit (*V*_u_) on the transfer volume, weight values can be specified relative to the transfer limit, such that they fall between 0 and 1.

To accommodate these constraints, the network is trained similarly to a self-organizing map,^51^ where weights are iteratively tuned to be more similar to input data. A benefit of training the weights on the inputs, rather than on the outputs of the activation function, is that the weights are independent of specific chemical conditions and only require the time to transition to be monotonic in initial catalyst concentration.

If a weight vector (*w⃑*_*i*_) is trained to identify class *i*, it should look more like the data (*x⃑*) from class *i* than a weight vector from any other class:

13 ‖*w⃑*_*i*_ − *x⃑*‖^2^ < ‖*w⃑*_j_ − *x⃑*‖^2^, ∀*j* ≠ *i*

where similarity is measured by the *L*^2^-norm: 
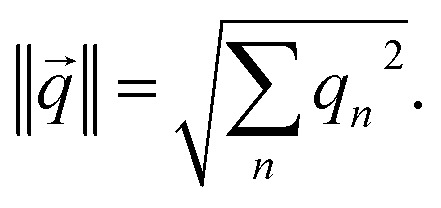
 This inequality can be summed across the remaining *K* − 1 classes to yield:14
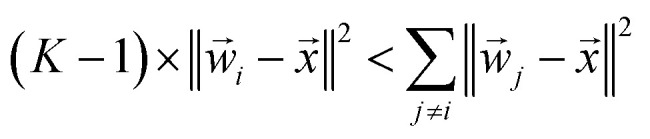
which can be rearranged to form the following loss function:15
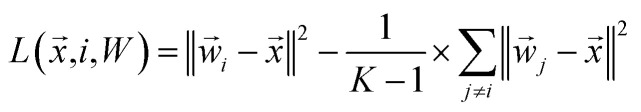
where *W* is the *K* × *N* matrix of all class weight vectors and *x⃑* is data from class *i*. Averaging the losses over the training data, we arrive at the following objective:16
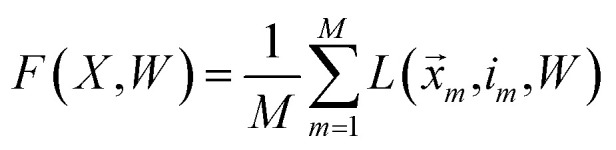
where *X* is the *M* × *N* matrix of training data and *i*_*m*_ is the class of the *m*^th^ training example (*x⃑*_*m*_).

We want to find the weight matrix (*W*) that minimizes this objective (*F*). Taking the partial derivative of the objective, for feature *n* and class *k*, yields:17
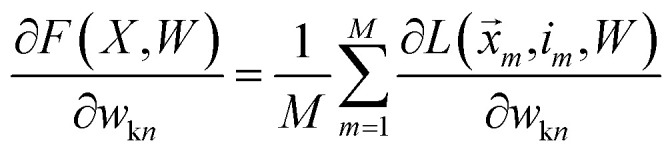
where18
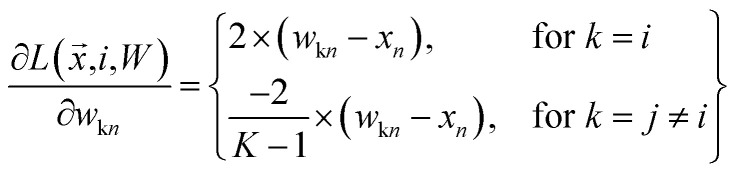
Using these derivatives, the optimal weights for each class are learned through gradient descent.^52^ After each descent step, the weights are constrained to the range of *w*_k*n*_ ∈ [0, 1] by clipping negative values to 0 and normalizing the remaining values by the class maximum. Since the weights are implemented as volume transfers, carried out on a fluid handler with a resolution of *V*_δ_ = 2.5 nL and a chosen transfer volume limit of 200 nL (*V*_u_), their experimental precision is approximately 6 bits (*V*_δ_/*V*_u_ = 80 levels).

A MATLAB implementation of the training routine is provided in the ESI (Listing S1†) and is also available on Github: github.com/Chris3Arcadia/AutocatalyticWTA. In addition to the experimentally tested network, one hundred 5-class WTA networks (see Fig. S10 and Table S1†) and one 9-class WTA network (see Fig. S11 and S12†) were trained and evaluated *in silico* to test the proposed classification scheme.

## Author contributions

C. E. A., A. D., K. O., and S.-L. C. performed experiments. C. E. A. and K. O. analyzed data. C. E. A and H. K. performed simulations. C. R., E. K., S. R., B. M. R., and J. K. R. provided direction and oversight. C. E. A., A. D., K. O., and J. K. R. drafted the paper. All authors provided notes and edits to the paper.

## Conflicts of interest

The authors declare that they have no conflicts of interest.

## Supplementary Material

SC-012-D0SC05860B-s001

## References

[cit1] Plasson R., Brandenburg A., Jullien L., Bersini H. (2011). J. Phys. Chem. A.

[cit2] Richard P. (2003). FEMS Microbiol. Rev..

[cit3] Thron C. (1996). Biophys. Chem..

[cit4] Thornberry N. A., Lazebnik Y. (1998). Science.

[cit5] Nies P. v., Westerlaken I., Blanken D., Salas M., Mencía M., Danelon C. (2018). Nat. Commun..

[cit6] Lee D. H., Severin K., Ghadiri M. R. (1997). Curr. Opin. Chem. Biol..

[cit7] Hordijk W., Hein J., Steel M. (2010). Entropy.

[cit8] Zador A. M. (2000). Nat. Neurosci..

[cit9] Simini F., 2016, arXiv:1602.09070

[cit10] Adleman L. (1994). Science.

[cit11] Carter F. L. (1984). Phys. D.

[cit12] Church G. M., Gao Y., Kosuri S. (2012). Science.

[cit13] Organick L., Ang S. D., Chen Y.-J., Lopez R., Yekhanin S., Makarychev K., Racz M. Z., Kamath G., Gopalan P., Nguyen B., Takahashi C. N., Newman S., Parker H.-Y., Rashtchian C., Stewart K., Gupta G., Carlson R., Mulligan J., Carmean D., Seelig G., Ceze L., Strauss K. (2018). Nat. Biotechnol..

[cit14] Anavy L., Vaknin I., Atar O., Amit R., Yakhini Z. (2019). Nat. Biotechnol..

[cit15] König N. F., Ouahabi A. A., Oswald L., Szweda R., Charles L., Lutz J.-F. (2019). Nat. Commun..

[cit16] Arcadia C. E., Kennedy E., Geiser J., Dombroski A., Oakley K., Chen S.-L., Sprague L., Ozmen M., Sello J., Weber P. M., Reda S., Rose C., Kim E., Rubenstein B. M., Rosenstein J. K. (2020). Nat. Commun..

[cit17] Rosenstein J. K., Dombroski A., Oakley K., Chen S. L., Tann H., Rubenstein B. M., Rose C., Reda S., Weber P. M., Kim E., Sello J., Geiser J., Kennedy E., Arcadia C. (2020). IEEE Trans. Nanobiosci..

[cit18] Hasty J., McMillen D., Collins J. J. (2002). Nature.

[cit19] Kim H., Bojar D., Fussenegger M. (2019). Proc. Natl. Acad. Sci. U. S. A..

[cit20] Qian L., Winfree E., Bruck J. (2011). Nature.

[cit21] Song X., Eshra A., Dwyer C., Reif J. (2017). RSC Adv..

[cit22] Cherry K. M., Qian L. (2018). Nature.

[cit23] Rambidi N. G. (1995). Biosystems.

[cit24] Gorecki J., Gizynski K., Guzowski J., Gorecka J. N., Garstecki P., Gruenert G., Dittrich P. (2015). Philos. Trans. R. Soc., A.

[cit25] Pandi A., Koch M., Voyvodic P. L., Soudier P., Bonnet J., Kushwaha M., Faulon J.-L. (2019). Nat. Commun..

[cit26] Arcadia C. E., Tann H., Dombroski A., Ferguson K., Chen S. L., Kim E., Rose C., Rubenstein B. M., Reda S., Rosenstein J. K. (2018). IEEE International Conference on Rebooting Computing.

[cit27] Brijder R. (2019). Nat. Comput..

[cit28] Blount D., Banda P., Teuscher C., Stefanovic D. (2017). Artif. Life.

[cit29] Rose C., Reda S., Rubenstein B. and Rosenstein J., 2018 IEEE International Symposium on Information Theory (ISIT), 2018, pp. 2236–2240

[cit30] Banda P., Teuscher C., Lakin M. R. (2013). Artif. Life.

[cit31] Banda P. and Teuscher C., Artificial Life Conference Proceedings, 2014, vol. 14, pp. 482–489

[cit32] LeCun Y., Bottou L., Bengio Y., Haffner P. (1998). Proc. IEEE.

[cit33] Steinfeld J. I., Francisco J. S. and Hase W. L., Chemical kinetics and dynamics, Prentice Hall Englewood Cliffs, New Jersey, 1989, vol. 3

[cit34] Kia B., Lindner J. F., Ditto W. L. (2017). Philos. Trans. R. Soc., A.

[cit35] Hinton G., Deng L., Yu D., Dahl G. E., Mohamed A., Jaitly N., Senior A., Vanhoucke V., Nguyen P., Sainath T. N., Kingsbury B. (2012). IEEE Signal Process. Mag..

[cit36] Nair V., Hinton G. E. (2010). ICML.

[cit37] Evans R. A. (2007). Aust. J. Chem..

[cit38] Kolb H. C., Finn M. G., Sharpless K. B. (2001). Angew. Chem., Int. Ed..

[cit39] Semenov S. N., Belding L., Cafferty B. J., Mousavi M. P., Finogenova A. M., Cruz R. S., Skorb E. V., Whitesides G. M. (2018). J. Am. Chem. Soc..

[cit40] Chan T. R., Hilgraf R., Sharpless K. B., Fokin V. V. (2004). Org. Lett..

[cit41] Döhler D., Michael P., Binder W. H. (2012). Macromolecules.

[cit42] Kaski S., Kohonen T. (1994). Neural Network..

[cit43] Maass W. (2006). Neural Comput..

[cit44] Marlin B., Swersky K., Chen B. and Freitas N., Proceedings of the Thirteenth International Conference on Artificial Intelligence and Statistics, Chia Laguna Resort, Sardinia, Italy, 2010, pp. 509–516

[cit45] Lipton R. (1995). Science.

[cit46] Yurke B., Turberfield A. J., Mills A. P., Simmel F. C., Neumann J. L. (2000). Nature.

[cit47] Woods D., Doty D., Myhrvold C., Hui J., Zhou F., Yin P., Winfree E. (2019). Nature.

[cit48] Mamet N., Harari G., Zamir A., Bachelet I. (2019). Comput. Biol. Chem..

[cit49] Gao R.-R., Yao T.-M., Lv X.-Y., Zhu Y.-Y., Zhang Y.-W., Shi S. (2017). Chem. Sci..

[cit50] Lin X., Yang S., Huang D., Guo C., Chen D., Yang Q., Li F. (2020). Chem. Sci..

[cit51] Kohonen T. (1990). Proc. IEEE.

[cit52] Ruder S., 2016, arXiv:1609.04747

