# Supplementary material for: Leveraging autocatalytic reactions for chemical domain image classification
Source: Chem Sci. 2021 Mar 3;12(15):5464–72. doi: 10.1039/d0sc05860b (PMC8179570; doi:10.1039/d0sc05860b)
Supplement: SC-012-D0SC05860B-s001 [file SC-012-D0SC05860B-s001.pdf]

Electronic Supplementary Material (ESI) for Chemical Science.
This journal is © The Royal Society of Chemistry 2021

Supplementary Information for

Leveraging autocatalytic reactions for chemical-domain image classification

Christopher E. Arcadia[a], Amanda Dombroski[b], Kady Oakley[b], Shui Ling Chen[b], Hokchhay Tann[a], Christopher Rose[a], Eunsuk Kim[b], Sherief Reda[a], Brenda M. Rubenstein[b], and Jacob K. Rosenstein*[a]

March 1, 2021

[a]School of Engineering, Brown University, Providence, RI, USA
[b]Department of Chemistry, Brown University, Providence, RI, USA

Contents

1 Code Repository **3**

2 Image Database **3**

3 Selected Classes **5**

4 Classifier Training **10**

5 Simulating Many 5-Class Networks **15**

6 Simulating a 9-Class Network **20**

1 Code Repository

All of the MATLAB methods and scripts used to train and simulate the autocatalysis-based winner-take-all networks can be found in the GitHub repository **AutocatalyticWTA**: `https://github.com/Chris3Arcadia/AutocatalyticWTA`.

2 Image Database

The images used in this study are from the CalTech 101 Silhouettes dataset[1], which itself is based on the CalTech 101 image annotations[2]. The database contains 8,641 binary 256-pixel (16×16) images that are each labeled with one of 101 object classes. The data available for each class is summarized by their averages in Figure S1. Additionally, as a measure of class distinctness, the Euclidean distance between each of the class-averaged images is shown in Figure S2. From both Figures S1 and S2, it is clear that many of the images contain class-specific features, but there are also several classes with significant feature overlap. In particular, large, filled circular objects, such as the soccer ball, stop sign, and yin yang symbol, are nearly indistinguishable.

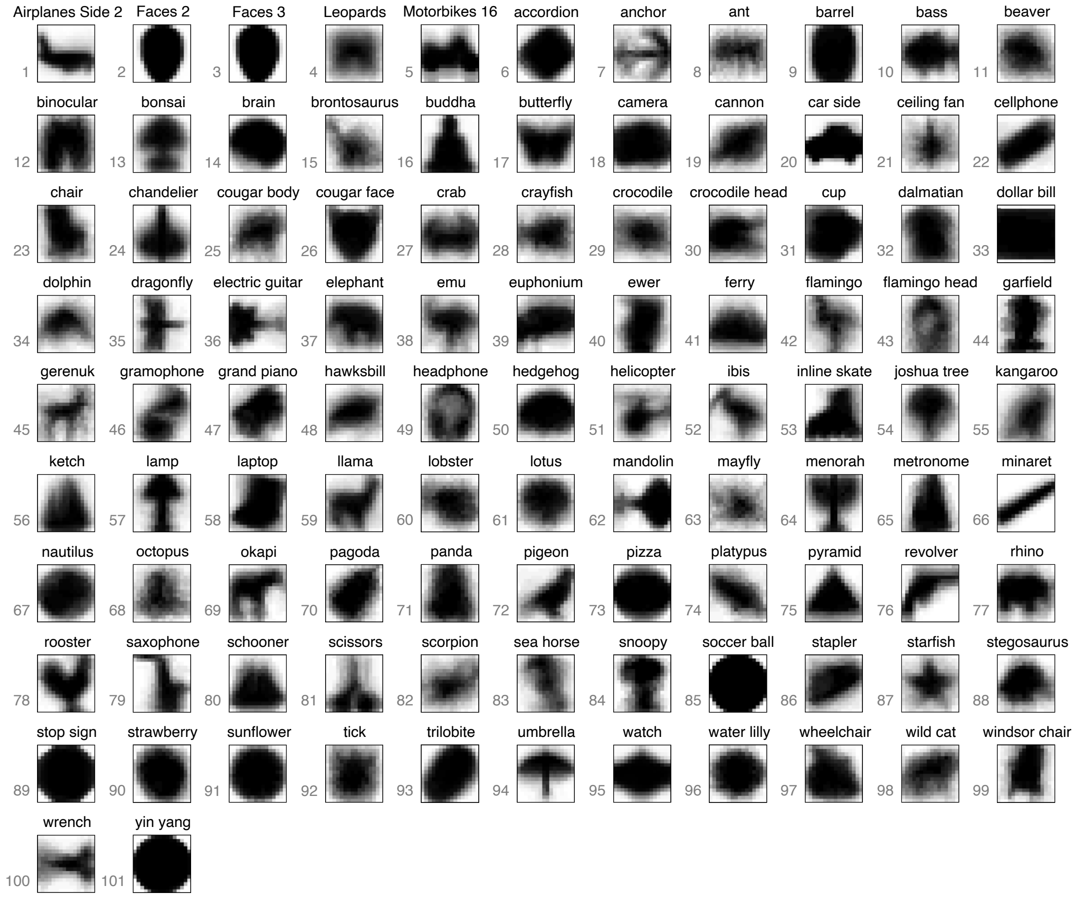

Figure S1 Averaged image data for each class in the CalTech 101 Silhouettes database[1]. Class indices and names are shown to the left of and above each image, respectively.

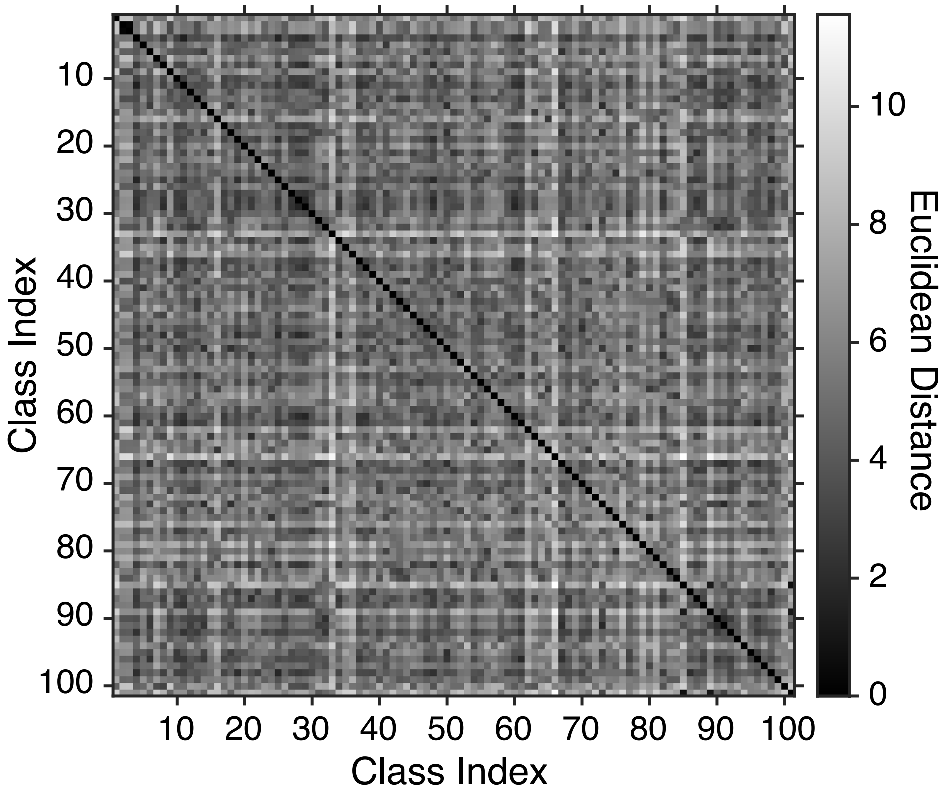

Figure S2 Euclidean distance between each class-averaged representation of the CalTech 101 Silhouettes[1] (those shown in Figure S1). Similar classes, such as Faces 2 and Faces 3 (indices 2 and 3, respectively), are nearer to each other and thus have intersections that appear darker in color. The distances (d) were computed as: $d(\vec{x},\vec{y}) = \sqrt{\sum_{i=1}^{256}(y_i - x_i)^2}$, where $\vec{x}$ and $\vec{y}$ are vectors formed by reshaping the 16$\times$16-pixel images being compared.

3 Selected Classes

The data used for training and testing our winner-take-all network is a subset of the images available in the CalTech 101 Silhouettes database[1]. Specifically, the dragonfly, ibis, kangaroo, llama, and starfish classes are used in the main text. For completeness, all of the images from these classes are displayed in Figures S3 (dragonfly), S4 (ibis), S5 (kangaroo), S6 (llama), and S7 (starfish). These classes were selected for aesthetic qualities, and to eliminate the large solid objects which would represent a much more difficult classification task.

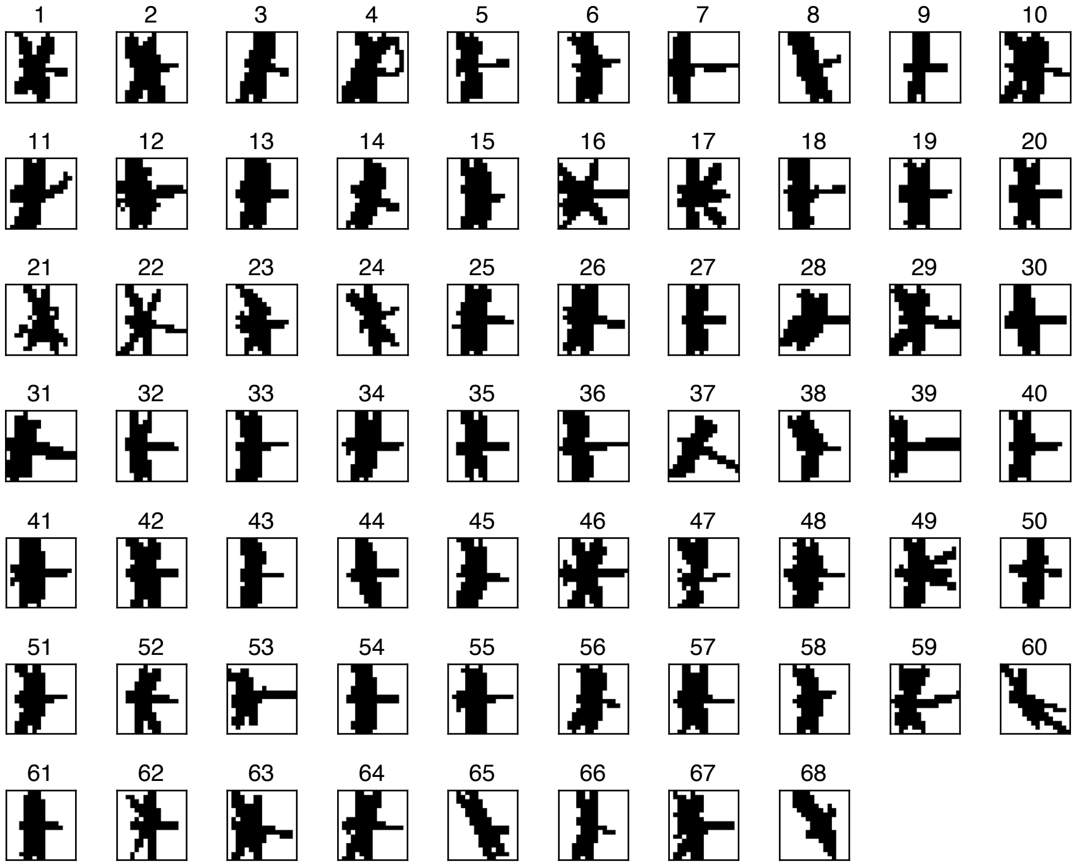

Figure S3 Dragonfly class images from the CalTech 101 Silhouettes database[1].

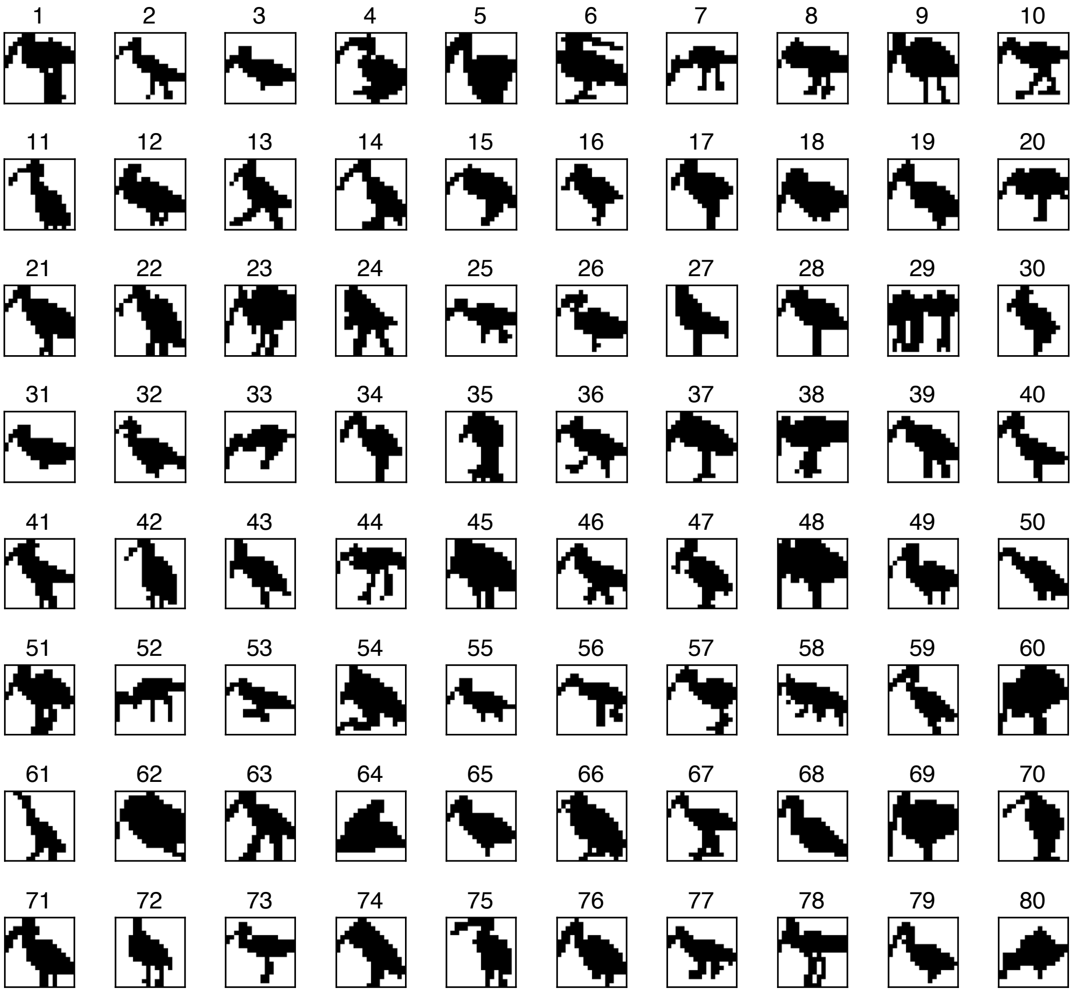

Figure S4 Ibis class images from the CalTech 101 Silhouettes database[1].

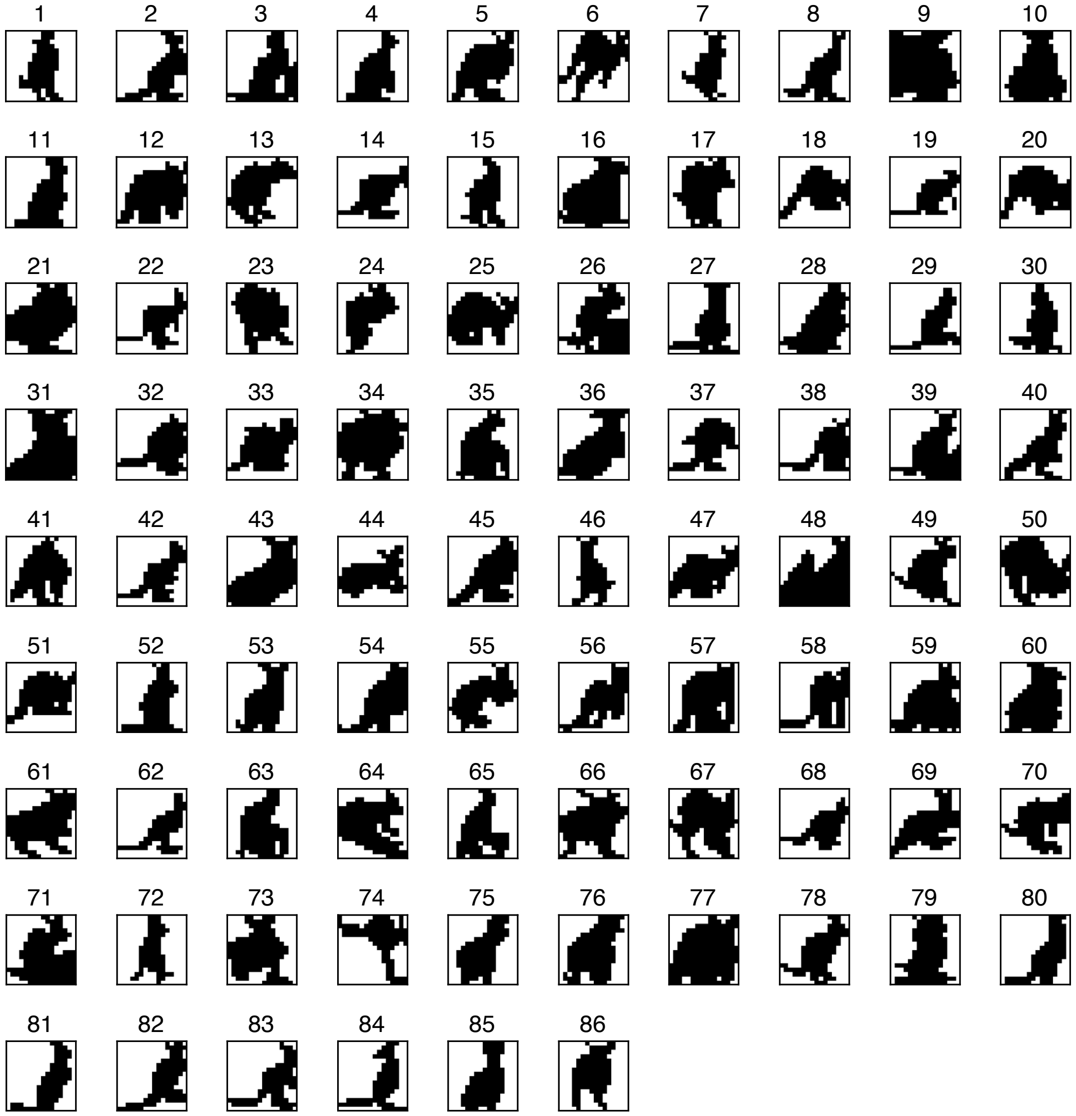

Figure S5 Kangaroo class images from the CalTech 101 Silhouettes database[1].

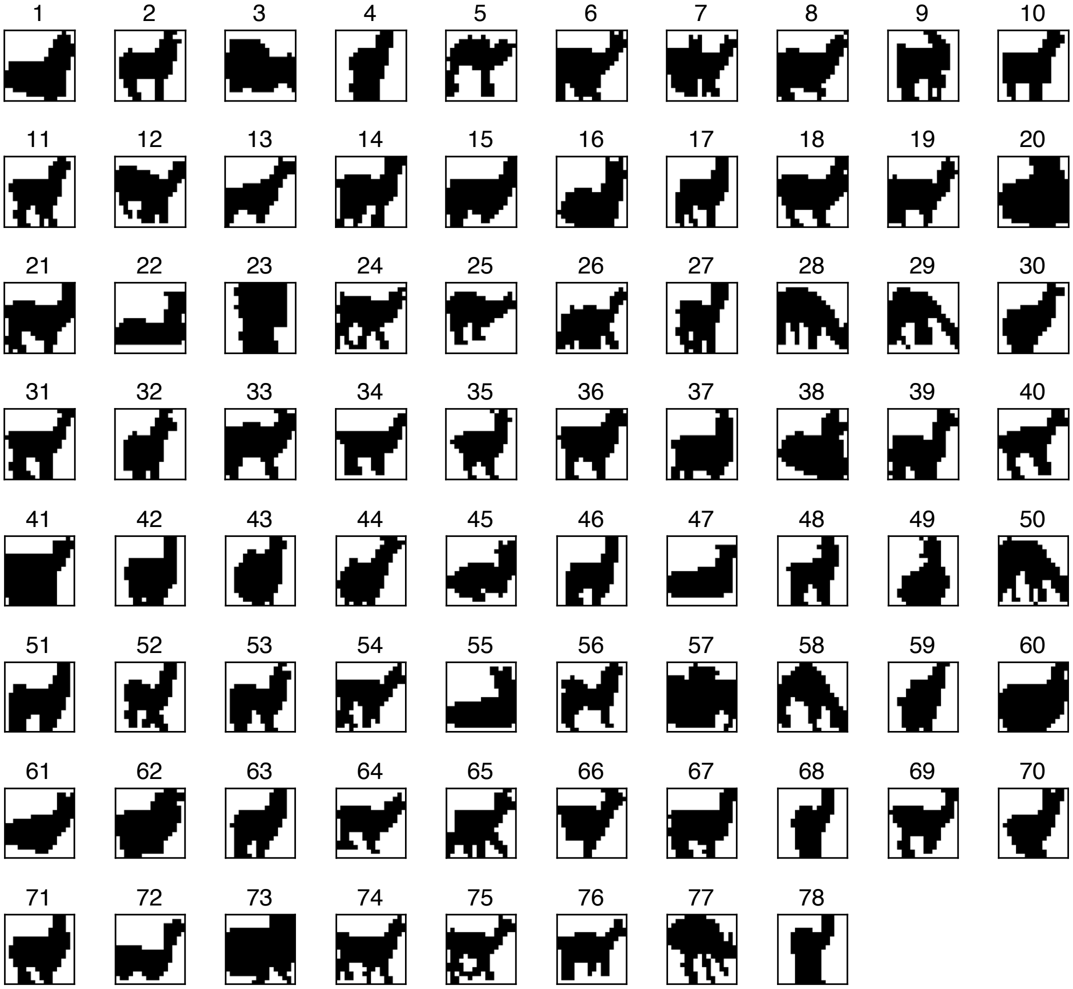

Figure S6 Llama class images from the CalTech 101 Silhouettes database[1].

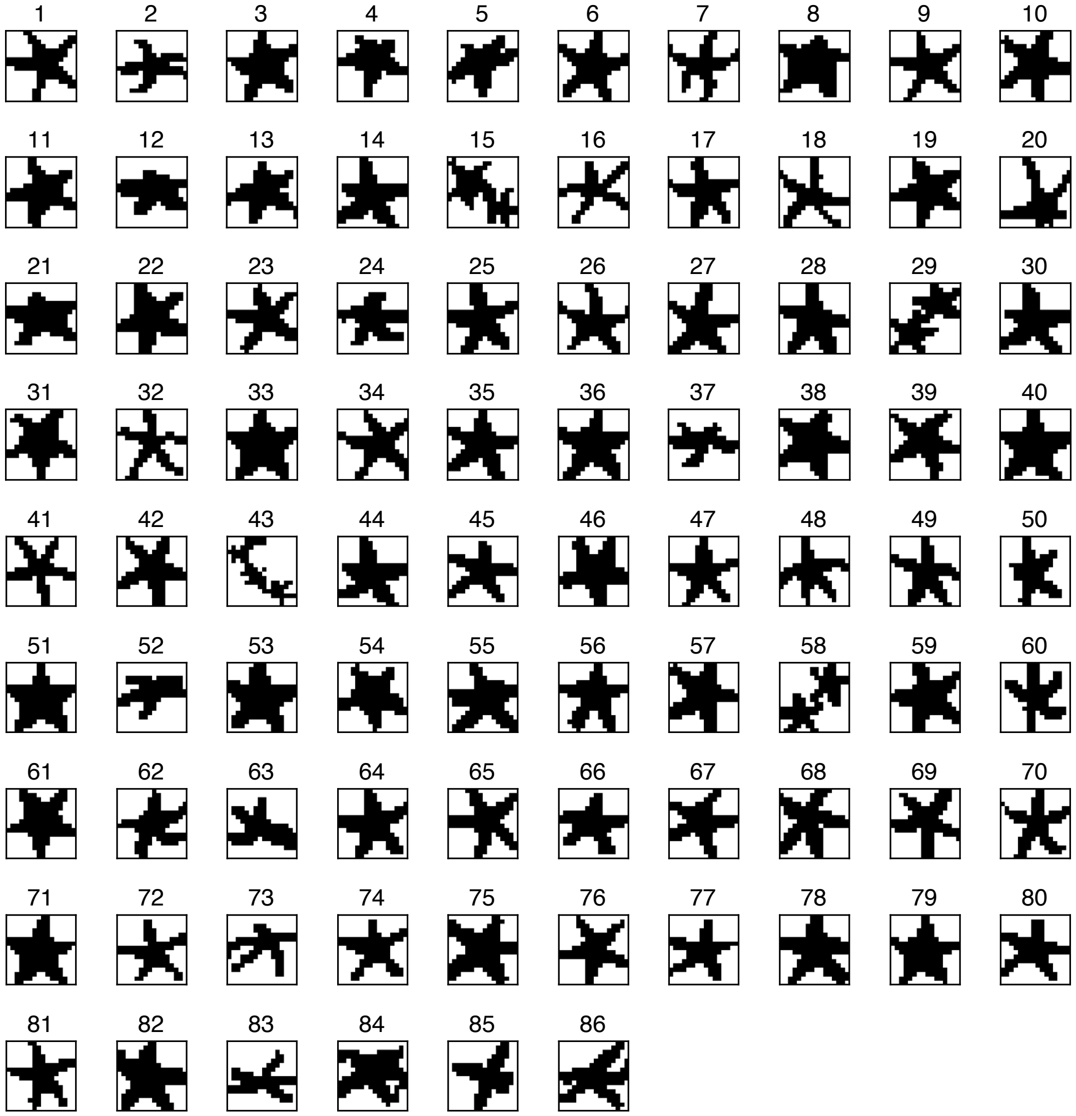

Figure S7 Starfish class images from the CalTech 101 Silhouettes database[1].

4 Classifier Training

The winner-take-all network in the main text was trained used gradient descent. Data from the 5 considered classes was split 70:30 into training (279 images) and test (119 images) sets. The objective function, F (defined in the main text), was minimized over 700 descent epochs using a learning rate of 5×10^{-4}. Since the partial derivatives of the objective, $\frac{\partial F}{\partial w_{kn}}$ (also defined in the main text), are dependent only on class-specific terms, the weight vectors for each class were updated separately during gradient descent. The exact MATLAB function used to tune the classifier weights is presented in Listing S1 (also see Section 1). After each epoch, the objective function was evaluated and its change between epochs was used to monitor training progress (see Figure S8a). The diminishing change in objective value towards the end of the training indicated a local minimum had been successfully reached (see Figure S8b). To assess classifier performance, all images from the considered classes were labeled by the trained network. A comparison between these predictions and the known true labels is shown in Figure S9. Ultimately, the network was found to have correctly classified 81.16% of all the data.

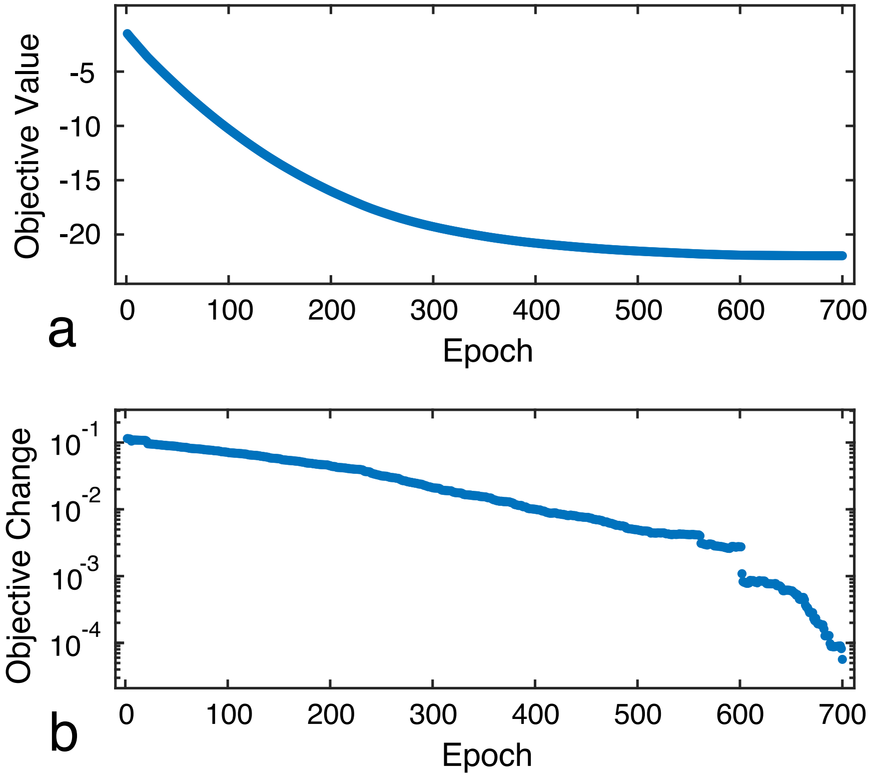

Figure S8 Training progress updates for the network shown in the main text. **a** Objective value (F_e) after each training epoch (e), as evaluated on the test set. **b** Absolute changes in objective value ($|F_e - F_{e-1}|$) over the course of training. The test set was composed of $Q = 119$ held out images (30% of the available data). Training was performed over the course of 700 epochs using a learning rate of 5×10^{-4}.

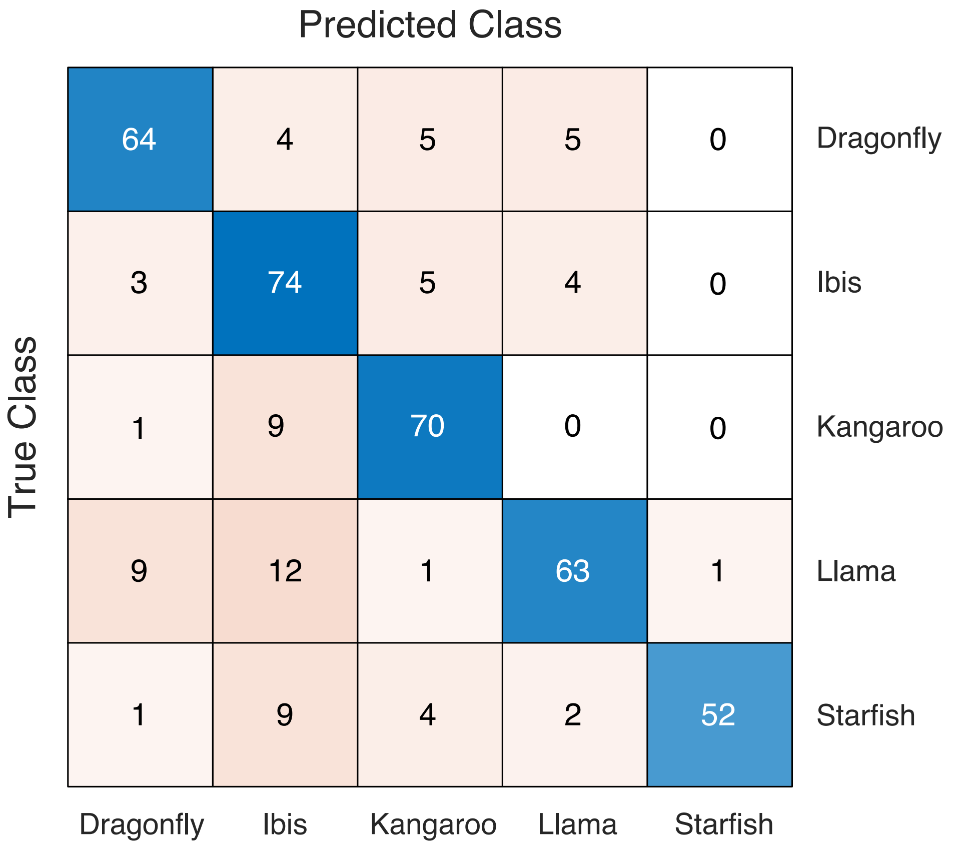

Figure S9 Simulated confusion matrix for the network shown in the main text. The values in the cells represent the number of images from each known class (row) that were labeled by each predicted class (column). The predicted class of each image was determined by the reaction well with the shortest simulated time to transition. Data from both the training and test sets was used to generate this matrix. Overall, classes were assigned with 81.16% accuracy.

Listing S1 Network training function, as implemented in MATLAB.

```
function [w,performance,training,testing] = trainer(x,y,w0,alpha,epoch,fraction,seed,verbose)
%%%%%%%%%%%%%%%%%%%%%%%%%%%%%%%%%%%%%%%%%%%%%%%%%%%%%%%%%%%%%%%%%%%%%%%%%%%%%%%%
% Train Winner-Take-All (WTA) Network
%
% Inputs:
%   x - training data (matrix with columns of training vectors) [Nf,Ne]
%   y - class labels (matrix with columns of training vectors) [1,Ne] (use integers 1,2,...)
%   w0 - the initial weight matrix [Nf,Nc]
%   alpha - the learning rate (for gradient descent)
%   epoch - the number of epochs
%   seed - seed used for random shuffling of training data (leave as [] if you do not wish to specify)
%   fraction - fraction of the total number of examples to use for training
%   verbose - boolean enabling detailed command line messages
%
% Outputs:
%   w - the final weight vector
%   performance - test performance (objective value) after each epoch
%   training - the indices of training data (numbered with respect to the output x and y)
%   testing - the indices of testing data (numbered with respect to the output x and y)
%
%%%%%%%%%%%%%%%%%%%%%%%%%%%%%%%%%%%%%%%%%%%%%%%%%%%%%%%%%%%%%%%%%%%%%%%%%%%%%%%%

% initialize weights
w = w0;
clear w0

% get shape of inputs
%  * Nf = number of features
%  * Ne = number of examples (for training, test, or validation)
%  * Nc = number of classes
[Nf,Ne] = size(x); % [Nf,Ne]
[~,Ne2] = size(y); %  [1,Ne]
[Nf2,Nc] = size(w); %  [Nf,Nc]
yUniq = unique(y);
Nc2 = length(yUniq);
if Ne~=Ne2 || Nf~=Nf2 || Nc~=Nc2
    error('Input data sizes are invalid.')
end
clear Ne2 Nf2 Nc2

% randomize training data
if isempty(seed)
    shuff.seed = rng;
else
    rng(seed);
```

```
    shuff.seed = seed;
end
shuff.perm = randperm(Ne);
x = x(:,shuff.perm);
y = y(shuff.perm);

% normalize the weights and training data
for c = 1:Nc
    w(:,c) = w(:,c)./(norm(w(:,c)));
end
xnorm = ones(size(x(1,:)));
for e = 1:Ne
    xnorm(e) = norm(x(:,e));
    x(:,e) = x(:,e)./xnorm(e);
end

% split data into testing and training sets
all = 1:Ne;
training = randperm(round(Ne*fraction));
testing = all;
% if there are examples left for testing
if length(training)~=length(all)
    testing(training) = [];
else
    % otherwise reuse training examples for testing (not advised)
end

% perform optimization of weights
sqrdL2diff = @(x,w) sum((w-x).^2); % squared L-2 norm of the difference
performance = zeros([epoch,length(testing)]); % track over all epochs
perfMeanCurr = 0;
% loop over each epoch
for n = 1:epoch
        % loop over each train example
        for e = training
            % find optimal weights for each class
            for c=1:Nc
                % perform gradient descent
                if yUniq(c)==y(e)
                    gradient = -2*(w(:,c)-x(:,e));
                else
                    gradient = (2/(Nc-1))*(w(:,c)-x(:,e));
                end
                w(:,c) = w(:,c) + alpha*gradient;

                % normalize weights to max
                w(:,c) = w(:,c)./(max(abs(w(:,c))));

                % clip off negative weights
                w(w(:,c)<0,c) = 0;
            end
        end
```

```
        counter = 1;
        % loop over each test example
        for e = testing
            % loop over each class
            xTest = xnorm(e)*x(:,e);
            for c=1:Nc
                % evaluate the portion of the objective function related to the current class
                if yUniq(c)==y(e)
                    factor = +1;
                else % yUniq(cc)==y(e)
                    factor = -1/(Nc-1);
                end
                performance(n,counter) = performance(n,counter) + factor*sqrdL2diff(xTest,w(:,c));
            end
            counter = counter + 1;
        end
        perfMeanLast = perfMeanCurr;
        perfMeanCurr = mean(performance(n,:));
    if verbose
        disp(['iteration: ' num2str(n) ', mean performance: ' sprintf('%0.4e',perfMeanCurr) ', change: ' sprintf('%0.4e',perfMeanCurr-perfMeanLast)]);
    end
end

% reference indices to the original data
training = shuff.perm(training);
testing = shuff.perm(testing);

end
```

5 Simulating Many 5-Class Networks

The winner-take-all network presented in the main text used a manually chosen subset of image classes from the the CalTech 101 Silhouettes dataset[1]. To see how similar networks would perform if the classes were randomly selected, we trained and simulated 100 such classifiers, each with their own unique set of 5 object classes. The results are summarized in Figure S10 and Table S1. Despite the known class degeneracies present in this dataset (see Section 3) and the simple network topology, the majority of classifiers perform significantly better than random guessing.

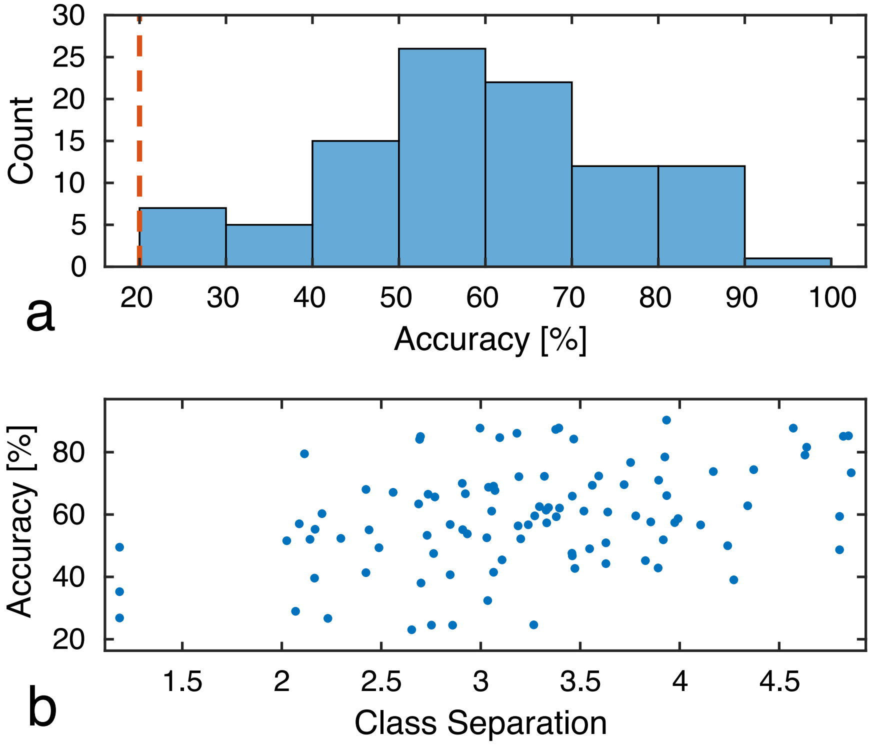

Figure S10 Overall classification accuracy across all 100 simulated 5-class networks. **a** A histogram of the classification accuracies observed across both the training and test sets (those shown in b). The dashed orange line represents the probability of randomly guessing the correct image class ($\frac{1}{5} = 20\%$). **b** Classification accuracies of the simulated networks plotted against their ensemble class separations, the minimal Euclidean distance between class averages (see Figure S2). There is a moderate positive correlation (Pearson coefficient of 0.39) between accuracy and class separation. Each network was randomly assigned a unique set of 5 image classes to learn (see Table S1) and was trained over 700 epochs with a learning rate of 5×10^{-4}.

Table S1 A summary of classification performance across all 100 of the simulated networks. The training, test, and overall classification accuracies are shown for each network, along with a list of the 5 associated classes and their ensemble class separation (Sep.).

#	Overall	Train	Test	Sep.	Classes
1	72.16%	73.96%	67.96%	3.19	Faces 3, windsor chair, euphonium, helicopter, brontosaurus
2	66.07%	65.23%	68.04%	3.93	rooster, scorpion, Faces 2, ketch, tick
3	67.71%	66.82%	69.79%	3.07	butterfly, beaver, barrel, nautilus, revolver
4	52.21%	56.32%	42.68%	3.20	ant, butterfly, cannon, scissors, pyramid
5	59.57%	54.55%	71.43%	3.27	elephant, gerenuk, strawberry, wild cat, dragonfly
6	41.36%	42.73%	38.17%	2.42	watch, scorpion, Leopards, ceiling fan, pizza
7	85.25%	88.24%	78.26%	4.85	ketch, car side, trilobite, mayfly, brain
8	46.75%	50.62%	37.68%	3.46	windsor chair, metronome, accordion, cup, garfield
9	84.11%	84.09%	84.15%	2.69	Airplanes Side 2, minaret, water lilly, kangaroo, brain
10	65.63%	64.84%	67.49%	2.77	bonsai, Faces 2, stapler, metronome, panda
11	79.07%	79.06%	79.10%	4.63	anchor, saxophone, Faces 2, butterfly, lamp
12	87.71%	86.76%	89.93%	4.57	brontosaurus, water lilly, windsor chair, Motorbikes 16, cellphone
13	39.60%	36.00%	48.00%	2.16	elephant, crocodile, cannon, cougar body, beaver
14	24.53%	26.11%	20.83%	2.75	brain, Leopards, scorpion, garfield, dolphin
15	24.51%	24.86%	23.68%	2.86	sunflower, platypus, yin yang, lobster, gerenuk
16	62.08%	63.64%	58.45%	3.40	ewer, ibis, cougar face, Leopards, mayfly
17	71.02%	72.73%	67.02%	3.89	minaret, ibis, barrel, brontosaurus, crayfish
18	57.02%	59.06%	52.29%	2.09	ketch, ferry, gerenuk, hedgehog, brain
19	26.69%	24.73%	31.25%	2.23	flamingo head, lobster, ferry, emu, soccer ball
20	90.29%	89.27%	92.70%	3.93	trilobite, Motorbikes 16, buddha, mandolin, scissors
21	79.47%	78.97%	80.63%	2.11	Leopards, wild cat, revolver, Airplanes Side 2, accordion
22	52.55%	55.31%	46.05%	3.03	pagoda, buddha, beaver, platypus, dollar bill
23	67.11%	67.94%	65.17%	2.56	chandelier, beaver, water lilly, wheelchair, rooster
24	66.50%	67.71%	63.71%	2.74	ibis, umbrella, menorah, starfish, scorpion
25	39.04%	37.25%	43.18%	4.27	soccer ball, panda, trilobite, crab, inline skate
26	28.96%	27.83%	31.63%	2.07	umbrella, platypus, hawksbill, strawberry, sunflower
27	53.79%	58.62%	42.53%	2.93	garfield, rhino, trilobite, cougar body, euphonium
28	56.37%	59.09%	50.00%	3.19	brain, ferry, pyramid, pigeon, gramophone

Table continues on the next page.

#	Overall	Train	Test	Sep.	Classes
29	60.29%	61.86%	56.63%	2.20	ceiling fan, schooner, pyramid, umbrella, octopus
30	55.27%	54.83%	56.29%	2.17	hedgehog, watch, crocodile, pyramid, chandelier
31	85.10%	87.67%	79.03%	4.82	inline skate, stapler, saxophone, rooster, brontosaurus
32	41.49%	41.59%	41.24%	3.06	joshua tree, menorah, hedgehog, cup, stop sign
33	65.91%	67.03%	63.29%	3.46	metronome, anchor, garfield, crayfish, kangaroo
34	84.21%	84.56%	83.38%	3.47	beaver, Motorbikes 16, stapler, ketch, ibis
35	78.46%	78.37%	78.68%	3.93	revolver, cougar face, watch, scissors, binocular
36	56.80%	57.81%	54.46%	2.85	buddha, sunflower, crocodile head, euphonium, bass
37	59.57%	59.79%	59.04%	3.78	ferry, emu, hedgehog, okapi, dragonfly
38	40.68%	40.76%	40.51%	2.85	euphonium, snoopy, pizza, sea horse, bass
39	59.31%	60.55%	56.41%	3.38	watch, euphonium, menorah, octopus, hawksbill
40	50.00%	47.03%	56.98%	4.24	saxophone, dollar bill, brontosaurus, grand piano, ferry
41	58.70%	58.80%	58.47%	3.99	metronome, soccer ball, Leopards, Faces 2, windsor chair
42	24.62%	23.08%	28.21%	3.27	pizza, beaver, windsor chair, yin yang, flamingo head
43	44.26%	44.93%	42.70%	3.63	stop sign, cup, snoopy, barrel, brain
44	26.83%	26.87%	26.74%	1.18	pagoda, soccer ball, pizza, stop sign, wheelchair
45	61.11%	58.94%	66.15%	3.52	saxophone, anchor, lobster, scissors, hedgehog
46	49.35%	49.07%	50.00%	2.49	laptop, helicopter, metronome, ferry, ceiling fan
47	60.81%	61.32%	59.62%	3.64	chandelier, dollar bill, binocular, menorah, laptop
48	87.72%	87.71%	87.76%	3.00	octopus, saxophone, Airplanes Side 2, tick, inline skate
49	61.09%	64.88%	52.27%	3.05	schooner, hedgehog, llama, wild cat, joshua tree
50	48.70%	51.06%	43.21%	4.80	saxophone, scissors, schooner, yin yang, cougar face
51	51.90%	49.22%	58.18%	3.92	soccer ball, starfish, laptop, elephant, minaret
52	69.38%	68.10%	72.36%	3.56	platypus, ewer, Faces 2, flamingo, anchor
53	68.05%	68.99%	65.85%	2.42	grand piano, mandolin, minaret, bonsai, joshua tree
54	85.03%	85.88%	83.05%	2.70	stegosaurus, Motorbikes 16, metronome, bass, scissors
55	56.73%	59.41%	50.49%	3.24	bonsai, dolphin, chair, rooster, panda
56	57.42%	56.22%	60.22%	3.97	laptop, pigeon, chandelier, headphone, wrench
57	52.35%	53.11%	50.56%	2.30	crocodile head, crab, dolphin, crocodile, cellphone
58	69.59%	71.76%	64.55%	3.72	ketch, windsor chair, crocodile head, llama, lotus
59	52.04%	53.37%	48.92%	2.14	lotus, rhino, llama, chair, Leopards
60	47.59%	47.78%	47.13%	3.46	pyramid, wheelchair, ant, elephant, dragonfly

Table continues on the next page.

#	Overall	Train	Test	Sep.	Classes
61	23.05%	22.71%	23.86%	2.65	accordion, helicopter, stapler, soccer ball, cannon
62	87.75%	86.78%	90.03%	3.39	cup, laptop, binocular, Airplanes Side 2, umbrella
63	49.03%	51.67%	42.86%	3.55	mandolin, camera, stapler, tick, crab
64	81.60%	80.26%	84.69%	4.64	cellphone, schooner, electric guitar, butterfly, wrench
65	70.00%	70.41%	69.05%	2.91	scorpion, dolphin, laptop, Faces 2, panda
66	50.93%	53.74%	44.33%	3.63	yin yang, cup, cellphone, emu, brain
67	61.42%	61.80%	60.53%	3.33	headphone, camera, pyramid, tick, wheelchair
68	76.69%	77.63%	74.49%	3.75	kangaroo, sea horse, dragonfly, ibis, strawberry
69	72.36%	72.44%	72.16%	3.59	stegosaurus, saxophone, grand piano, revolver, ant
70	57.63%	58.47%	55.70%	3.85	cougar face, wheelchair, saxophone, emu, brontosaurus
71	32.41%	33.99%	28.74%	3.04	joshua tree, crab, cup, wrench, lamp
72	55.08%	55.14%	54.95%	2.44	emu, joshua tree, crayfish, barrel, crab
73	42.86%	43.98%	40.24%	3.89	laptop, strawberry, cougar body, chair, gramophone
74	51.60%	54.31%	45.24%	2.02	crayfish, panda, buddha, lobster, ceiling fan
75	73.39%	74.11%	71.71%	4.86	ibis, wrench, ferry, dragonfly, Faces 2
76	42.72%	42.13%	44.09%	3.47	pizza, flamingo head, panda, menorah, starfish
77	49.52%	49.54%	49.46%	1.18	soccer ball, dollar bill, chair, laptop, stop sign
78	35.25%	36.71%	31.82%	1.18	ferry, stop sign, crayfish, soccer ball, wild cat
79	74.39%	76.73%	68.97%	4.37	inline skate, headphone, umbrella, chandelier, platypus
80	55.15%	57.58%	49.49%	2.91	nautilus, car side, tick, okapi, joshua tree
81	72.26%	74.51%	67.05%	3.32	llama, metronome, kangaroo, saxophone, windsor chair
82	45.22%	48.42%	37.80%	3.83	helicopter, cougar face, gerenuk, metronome, crocodile head
83	53.33%	52.51%	55.26%	2.73	octopus, stapler, minaret, cellphone, saxophone
84	56.67%	62.50%	43.06%	4.11	platypus, panda, nautilus, llama, snoopy
85	45.45%	46.95%	41.96%	3.11	grand piano, car side, water lilly, crocodile head, stop sign
86	69.10%	71.67%	63.11%	3.06	windsor chair, crocodile head, buddha, menorah, joshua tree
87	57.33%	58.14%	55.43%	3.33	scorpion, cougar face, inline skate, wheelchair, dalmatian
88	68.75%	69.86%	66.13%	3.04	binocular, windsor chair, inline skate, strawberry, cup
89	73.79%	75.93%	68.82%	4.17	rhino, accordion, electric guitar, wheelchair, lamp
90	62.23%	61.50%	63.92%	3.34	wheelchair, llama, lotus, cougar face, pizza
91	38.03%	38.93%	35.94%	2.70	crocodile, ceiling fan, snoopy, wild cat, camera
92	87.29%	89.02%	83.23%	3.38	Motorbikes 16, cup, panda, trilobite, laptop

Table continues on the next page.

#	Overall	Train	Test	Sep.	Classes
93	59.40%	60.37%	57.14%	4.80	hedgehog, schooner, scissors, saxophone, wrench
94	63.40%	62.31%	65.94%	2.69	wrench, cougar face, emu, lotus, watch
95	86.07%	88.05%	81.48%	3.18	electric guitar, windsor chair, ketch, platypus, ibis
96	47.50%	47.77%	46.88%	2.76	cougar face, rooster, butterfly, bass, rhino
97	62.81%	64.46%	58.97%	4.34	electric guitar, watch, sea horse, menorah, dolphin
98	62.54%	63.27%	60.82%	3.29	hedgehog, pyramid, gramophone, cougar body, ketch
99	66.67%	67.31%	65.15%	2.92	electric guitar, bass, pigeon, Leopards, flamingo
100	84.67%	86.05%	81.43%	3.10	Motorbikes 16, crab, crocodile head, garfield, dragonfly

6 Simulating a 9-Class Network

To demonstrate that an autocatalytic winner-take-all network can be successfully applied to more difficult classification tasks, we trained one such network over 900 epochs on the following 9 classes from the Caltech 101 Silhouettes database[1]: revolver, lamp, mandolin, headphone, umbrella, helicopter, pyramid, chair, saxophone. The learned weights are shown in Figure S11. These weights were used to classify all images associated with the 9 considered classes. The resulting confusion matrix is shown in Figure S12, and the overall classification accuracy was 80.00%.

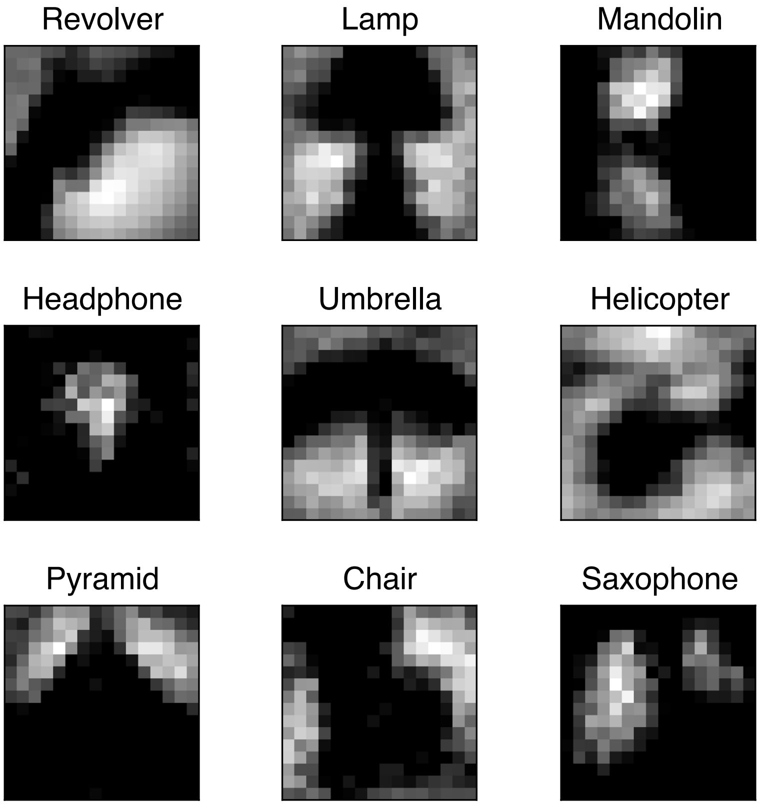

Figure S11 Classifier weights for the 9-class network. Weights were learned over the course of 900 gradient descent epochs with a learning rate of 5×10^{-4}.

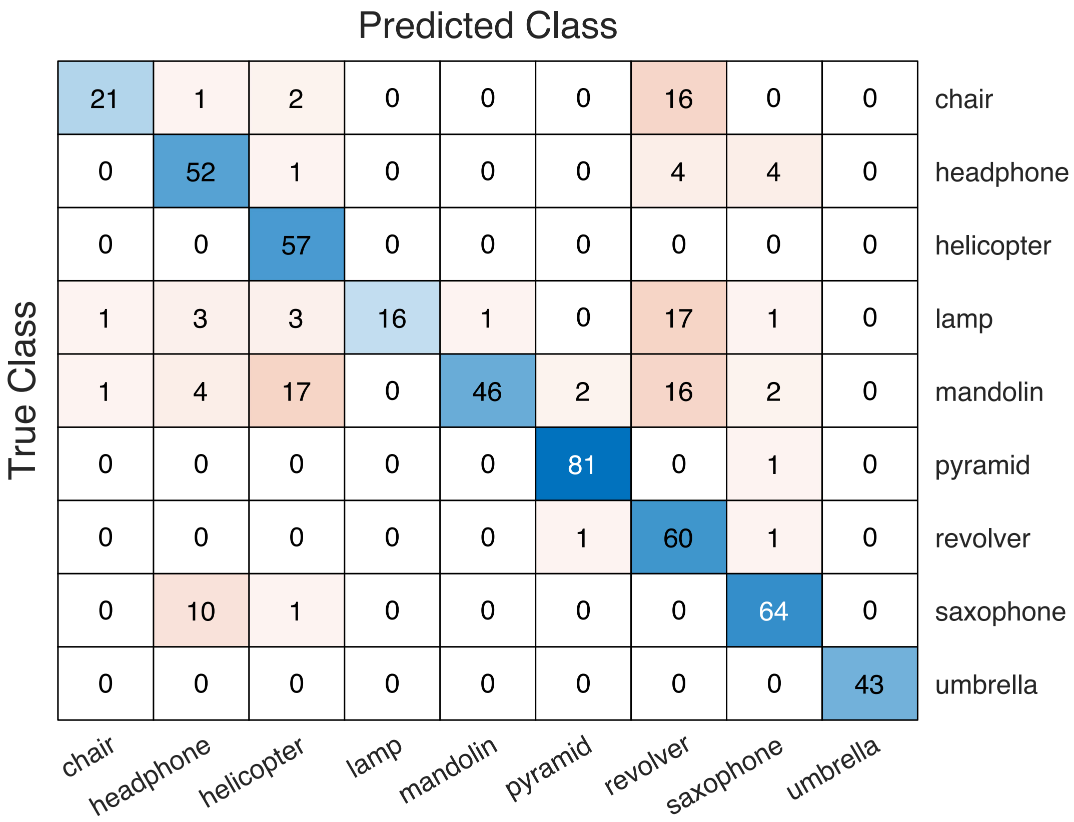

Figure S12 Classification confusion matrix for the 9-class network. Images from both the training and test sets were included. Overall, classes were assigned with 80.00% accuracy.

Notes and references

[1] B. Marlin, K. Swersky, B. Chen and N. Freitas, Proceedings of the Thirteenth International Conference on Artificial Intelligence and Statistics, Chia Laguna Resort, Sardinia, Italy, 2010, pp. 509–516.

[2] L. Fei-Fei, R. Fergus and P. Perona, *IEEE Transactions on Pattern Analysis and Machine Intelligence*, 2006, **28**, 594–611.
